# Structural basis for tunable control of actin dynamics by myosin-15 in mechanosensory stereocilia

**DOI:** 10.1126/sciadv.abl4733

**Published:** 2022-07-20

**Authors:** Rui Gong, Fangfang Jiang, Zane G. Moreland, Matthew J. Reynolds, Santiago Espinosa de los Reyes, Pinar Gurel, Arik Shams, James B. Heidings, Michael R. Bowl, Jonathan E. Bird, Gregory M. Alushin

**Affiliations:** ^1^ Laboratory of Structural Biophysics and Mechanobiology, The Rockefeller University, New York, NY, USA.; ^2^ Department of Pharmacology and Therapeutics, University of Florida, Gainesville, FL, USA.; ^3^ Laboratory of Molecular Genetics, National Institute on Deafness and Other Communication Disorders, National Institutes of Health, Bethesda, MD, USA.; ^4^ Mammalian Genetics Unit, MRC Harwell Institute, Harwell Campus, Oxfordshire, UK.; ^5^ UCL Ear Institute, University College London, London, UK.

## Abstract

The motor protein myosin-15 is necessary for the development and maintenance of mechanosensory stereocilia, and mutations in myosin-15 cause hereditary deafness. In addition to transporting actin regulatory machinery to stereocilia tips, myosin-15 directly nucleates actin filament (“F-actin”) assembly, which is disrupted by a progressive hearing loss mutation (p.D1647G, “*jordan*”). Here, we present cryo–electron microscopy structures of myosin-15 bound to F-actin, providing a framework for interpreting the impacts of deafness mutations on motor activity and actin nucleation. Rigor myosin-15 evokes conformational changes in F-actin yet maintains flexibility in actin’s D-loop, which mediates inter-subunit contacts, while the *jordan* mutant locks the D-loop in a single conformation. Adenosine diphosphate–bound myosin-15 also locks the D-loop, which correspondingly blunts actin-polymerization stimulation. We propose myosin-15 enhances polymerization by bridging actin protomers, regulating nucleation efficiency by modulating actin’s structural plasticity in a myosin nucleotide state–dependent manner. This tunable regulation of actin polymerization could be harnessed to precisely control stereocilium height.

## Teaser

Cryo-EM structures of myosin-15 bound to F-actin provide insights into its molecular motor and actin nucleation mechanisms.

## INTRODUCTION

The stereocilia of inner ear hair cells are rod-like membrane protrusions responsible for sound detection. Each hair cell assembles a staircase-shaped mechanosensory hair bundle during development, which contains three rows of stereocilia of increasing height. Stereocilia are primarily composed of parallel bundles of filamentous actin (F-actin) cross-linked to form a paracrystalline array (*1*). Stereocilia develop from microvilli that undergo an exquisite morphogenetic program characterized by F-actin elongation and bundling, presumed to be orchestrated by coordinated actin polymerization and bundling protein engagement (*1*). Once a stereocilium is established, slow actin dynamics maintain its architecture throughout adult life (*2*). Finely tuned regulation of actin dynamics is critical for stereocilium height determination, and mutations in actin and its regulatory factors are frequently associated with hearing loss (*3*). Nevertheless, the molecular mechanisms that precisely define the height of individual stereocilia during development and maintain them for a lifetime remain elusive.

Unconventional myosin-15 (encoded by the *Myo15a* gene in mouse and *MYO15A* in humans) is a plus-end directed molecular motor essential for stereocilia development and maintenance (*4*–*7*). More than 300 mutations have been mapped to the human *MYO15A* gene that cause autosomal recessive nonsyndromic deafness, DFNB3 (*7*, *8*). Within hair cells, myosin-15 localizes to the distal tips of stereocilia, the major sites of actin turnover that are enriched with F-actin plus (“barbed”) ends (*2*, *6*, *9*). In mice homozygous for the *Myo15a* “*shaker 2*” allele (p.C1779Y), myosin-15 trafficking is disrupted in nascent stereocilia (*9*), resulting in the failure of stereocilia to elongate and ultimately causing profound hearing loss (*10*). Myosin-15 transports whirlin (WHRN), epidermal growth factor receptor pathway substrate 8 (EPS8), G protein signaling modulator 2 (GPSM2), and G protein subunit alpha I3 (GNAI3) to stereocilia tips, an “elongation network” of interacting proteins required for stereocilium biogenesis, each member of which phenocopies *shaker 2* when individually deleted in mice (*6*, *11*–*13*). While WHRN, GPSM2, and GNAI3 currently have no known elongation network functions beyond mediating protein-protein interactions, EPS8 is an established actin-binding protein (ABP) that exhibits filament bundling and plus-end capping activity (*14*). It has therefore been proposed that myosin-15 mediates stereocilia growth by transporting elongation network components to the tip compartment, where they serve as the regulatory machinery governing F-actin assembly (*6*, *12*, *13*). Nevertheless, the functional consequences of specific myosin-15 mutations remain poorly understood because of the absence of structural data and limited functional characterization of the myosin-15 protein.

**Fig. 1.**
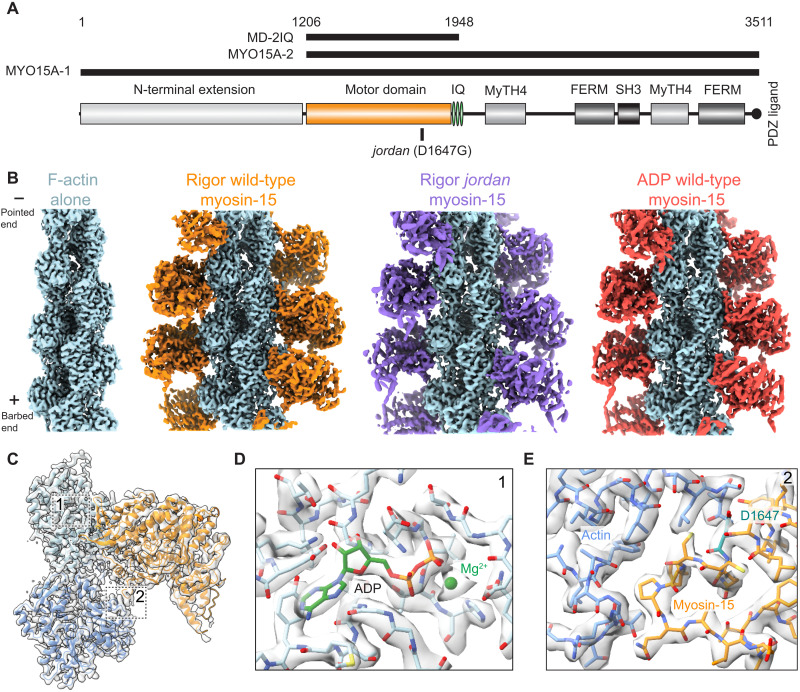
Cryo–electron microscopy structures of actomyosin-15 complexes. (**A**) Diagram of *Mus musculus* myosin-15 primary domain structure. (**B**) Cryo–electron microscopy (cryo-EM) maps of F-actin alone and indicated actomyosin-15 complexes. (**C**) Cryo-EM map and ribbon diagram of two actin and one myosin-15 subunits from the rigor wild-type actomyosin-15 reconstruction. Numbered boxes correspond to detail views in subsequent panels. (**D**) Cryo-EM map and stick models around actin’s ADP binding pocket. (**E**) Cryo-EM map and stick models at the actin-myosin loop 3 interface. The position of the *jordan* mutation (D1647) is highlighted in teal.

In addition to its canonical adenosine triphosphatase (ATPase) motor domain, myosin-15 contains myosin tail homology 4 (MyTH4), band 4.1, ezrin, radixin, moesin (FERM), and Src homology 3 (SH3) domains (Fig. 1A), structural elements that mediate protein-protein interactions with cytoskeletal partners and cargos. These domains are also found in the related stereocilia, microvilli, and filopodia-associated motors myosin-7 and myosin-10, which, along with myosin-15, constitute the MyTH4-FERM family of myosins (*15*). The myosin-15 protein is expressed as two alternatively spliced isoforms that solely differ by a unique 133-kDa proline-rich N-terminal extension containing no predicted domains (Fig. 1A). The shorter MYO15A-2 isoform is critical for stereocilia biogenesis and is sufficient for elongation network transport, while the longer MYO15A-1 isoform is specifically required to maintain the F-actin cores of the shorter mechanotransducing stereocilia rows, suggesting that the N-terminal extension promotes cytoskeletal integrity through unknown mechanisms (*5*). The MYO15A motor domain has a high duty ratio, spending at least 50% of its mechanochemical cycle bound to the actin filament (*4*, *16*). This is facilitated by the motor’s substantial F-actin binding affinity when occupied by adenosine diphosphate (ADP) [*K*_DA_ (actin-binding dissociation constant in the presence of saturating ADP) = 310 nM versus *K*_A_ (actin-binding dissociation constant in the absence of nucleotide) = 200 nM] and ADP release being the slowest step in its nucleotide hydrolysis cycle (12 s^−1^), attributes that restrict the motor’s adenosine triphosphate (ATP) binding–coupled dissociation from F-actin (*16*). Similar properties are hallmarks of other well-characterized unconventional myosins whose F-actin engagement is modulated by force to regulate processive cargo trafficking (myosin-5a and myosin-6) and cytoskeleton-membrane tethering (myosin-1b) functions (*17*, *18*). Biophysical (*19*, *20*) and structural (*21*–*23*) studies of these myosins have revealed coupling between nucleotide binding pocket rearrangements enabling ADP release and minor lever-arm swings of differing magnitude and directionality, facilitating tuned mechanical gating of ADP release/ATP rebinding through their lever arms to mediate force-sensitive F-actin dissociation (*24*). By analogy to these motors, myosin-15 has also been speculated to be force sensitive (*16*), but this has not been experimentally examined and it remains unclear whether mechanical gating of ADP release through this mechanism is a universal feature of myosins. Furthermore, the structural mechanisms mediating the weak thermodynamic coupling between myosin-15’s ADP binding and F-actin engagement remain unknown.

In a companion study, we identified the myosin-15 missense mutation p.D1647G, hereafter referred to as the *jordan* mutant (Fig. 1A), in a forward genetic screen for lesions leading to progressive hearing loss in mice (*25*). The mutant allele was named after the first deaf president of Gallaudet University. Hair cell stereocilia in the *jordan* mouse are developmentally stunted, and the short stereocilia actin cores additionally retract during aging (*25*). Although its actin-activated ATPase activity (wild type, *k*_cat_ = 5.8 s^−1^; *jordan*, *k*_cat_ = 0.87 s^−1^) and F-actin binding affinity (wild type, *k*_ATPase_ = 29.1 μM; *jordan*, *k*_ATPase_ = 114.3 μM) are somewhat compromised, purified *jordan* myosin-15 is nevertheless an active motor, and WHRN, EPS8, GPSM2 and GNAI3 localize to the tips of developing stereocilia in the *jordan* mouse, leading us to hypothesize that myosin-15 could control stereocilium height through additional mechanisms. Consistently, we found that a minimal motor domain truncation of myosin-15 (S1-like fragment) markedly stimulates actin polymerization in vitro, with the nucleotide-free “rigor” state specifically acting as a nucleation factor, while the *jordan* mutant instead suppresses actin assembly [figure 6 in (*25*)]. Early studies of the S1 fragment of skeletal muscle myosin demonstrated its capacity to nucleate actin polymerization in vitro (*26*–*28*), but a physiological role for this activity has not, to our knowledge, been reported in vivo. Our studies suggest that in addition to trafficking proteins, myosin-15 directly regulates actin dynamics required for stereocilia growth. However, the underlying molecular mechanism, and how it is disrupted by a single amino acid substitution in the *jordan* mutant, remains unknown.

Actin polymerization occurs through two fundamental processes: “nucleation,” where free subunits self-assemble into a minimal polymerization-competent unit (an energetically unfavorable process requiring a substantial energy barrier be overcome), and “elongation,” where additional free subunits are added to the end of a preexisting filament (an energetically favorable process), thereby extending it (*29*). In addition, actin polymerization is coupled to subunit ATP hydrolysis, which provides the driving force for dynamic actin assembly and disassembly in the cell. Each actin monomer binds ATP, which is rapidly hydrolyzed by subunits upon their incorporation into a filament, followed by slower phosphate release. This results in metastable aged ADP F-actin whose depolymerization is thermodynamically favored (*29*). As the intrinsic polymerization and depolymerization kinetics of actin are slow, both assembly processes, as well as F-actin disassembly, are tightly regulated by dozens of ABPs, including severing proteins, capping proteins, processive polymerases, and nucleators, whose activities must be precisely coordinated to construct elaborated cellular protrusions such as stereocilia (*1*, *29*). These ABPs frequently use their actin-binding interfaces to modulate G-actin monomer/F-actin protomer conformation and thereby fine-tune actin polymerization dynamics (*29*). Within actin subdomain 2, the structurally polymorphic “D-loop” plays a prominent role in this conformational regulation (*30*–*32*). The D-loop undergoes a substantial rearrangement during actin polymerization, enabling it to form the major longitudinal contact between actin protomers in the filament (*33*). In F-actin, distinct D-loop conformations have been visualized in the presence of bound ABPs (*21*, *22*, *34*, *35*), toxins (*36*, *37*), and biochemical mimics of actin nucleotide states present as intermediates during polymerization (*36*, *38*), highlighting this flexible element as a major site of F-actin structural regulation. Correspondingly, several myosin motors have been reported to modulate actin’s conformation through direct contacts with the D-loop upon F-actin binding (*21*–*23*, *34*), and distinct actin conformations were visualized for the Mg-ADP and rigor stages of myosin-6’s mechanochemical cycle, mediated by the motor’s contact with the D-loop (*22*). This led us to hypothesize that reciprocal conformational changes at the actin-myosin interface could be harnessed by myosin-15 during its mechanochemical cycle to precisely regulate F-actin polymerization dynamics to control stereocilium height.

Here, we report the cryo–electron microscopy (cryo-EM) structure of the rigor wild-type myosin-15 motor domain bound to ADP F-actin at 2.84-Å resolution, providing a framework for broadly interpreting deafness mutations in the motor domain and at the actin-myosin interface. To probe myosin-15’s mechanochemical cycle and the mechanisms by which it regulates F-actin assembly, we also obtained cryo-EM structures of ADP F-actin alone (2.82 Å), as well as rigor *jordan* myosin-15 (3.76 Å) and Mg-ADP wild-type myosin-15 (3.63 Å) bound to F-actin. Our F-actin alone structure reveals a region of the D-loop (residues G48-Q49) that is structurally flexible but nevertheless primarily adopts a mixture of two conformations. Rigor wild-type myosin-15 engagement elicits rearrangements at the actin-myosin interface that are propagated through the actin structure; however, the D-loop continues to adopt two conformations. Binding of the *jordan* deafness mutant evokes similar overall rearrangements but notably locks the D-loop in a single conformation, as does Mg-ADP wild-type myosin-15, which shows reduced F-actin stimulation relative to the rigor state in polymerization assays. Furthermore, our Mg-ADP–bound actomyosin-15 structure reveals minimal rearrangements in the motor domain and lever arm when compared to the rigor state, providing a structural rationale for myosin-15’s high actin-binding affinity in the presence of ADP and suggesting that, unlike other high-duty ratio motors, ADP release is unlikely to be mechanically gated in myosin-15. Our studies suggest that strongly bound myosin-15 promotes F-actin assembly by reinforcing longitudinal contacts between actin protomers with a nucleation efficiency controlled by the motor domain’s nucleotide state, which modulates both the motor’s actin-binding affinity and actin’s internal conformational flexibility, notably in a region that undergoes rearrangements during polymerization. We propose that this tunable direct regulation of actin assembly dynamics facilitates precise control of stereocilia height by myosin-15 in vivo, which is completely compromised by the *jordan* deafness mutation.

## RESULTS

### Cryo-EM reconstructions of actomyosin-15 complexes and bare F-actin

Using cryo-EM and the iterative-real space helical symmetry reconstruction (IHRSR) procedure as implemented in RELION-3 (*39*, *40*), we determined structures of a wild-type mouse myosin-15 construct containing the motor domain and 2 IQ motifs (*4*) (“MD-2IQ”; Fig. 1A) bound to ADP chicken skeletal muscle F-actin at a 1:1 stoichiometry in the rigor and Mg-ADP states at an average resolution of 2.84 and 3.63 Å, respectively (see Materials and Methods and table S1). We additionally determined the structure of the *jordan* deafness mutant bound to F-actin at 3.76-Å resolution (Fig. 1B and fig. S1, A and B). The sub–3-Å resolution of our rigor wild-type structure facilitated building an accurate atomic model of the myosin-15 motor domain (Fig. 1C), which has not, to our knowledge, previously been structurally visualized in the absence or presence of F-actin.

As has been reported in other cryo-EM studies of actomyosin (*21*, *22*, *34*, *41*), the local resolution of these reconstructions is highest in the center of the filament and decreases radially outward toward the distal tip of the myosin lever arm (fig. S1B). For all three complexes, the resolution of F-actin and the actin-myosin interface extends beyond 3.5 Å, yielding models with unambiguous side-chain density within these areas (Fig. 1, D and E, and fig. S1B). Most side chains in the motor domain are clearly resolved, and secondary structures can be identified in and around the distal converter domain (Fig. 1E and fig. S1B). To visualize structural changes evoked in F-actin by myosin-15, we also reconstructed bare ADP F-actin at a global resolution of 2.82 Å (Fig. 1B and fig. S1, A and B), to our knowledge the highest resolution mammalian F-actin structure reported to date. This density map facilitated direct model building and refinement of actin’s bound Mg-ADP and all actin residues except the first five at the protein’s flexible N terminus. Although stereocilia contain the cytoplasmic β- and γ-isoforms of actin rather than the skeletal muscle α-isoform used here, all actin isoforms are at least 93% identical to one another (*42*), with most differences in the flexible, negatively charged N terminus of the protein. Previous actomyosin reconstructions featuring cardiac α-actin (*41*) and cytoplasmic γ-actin (*34*) revealed no notable actin isoform–specific structural idiosyncrasies, and the residues found at the actin-myosin interface are identical between isoforms (*42*), including all specific interactions discussed in this paper.

### Structural interpretation of deafness-causing myosin-15 mutations

**Fig. 2.**
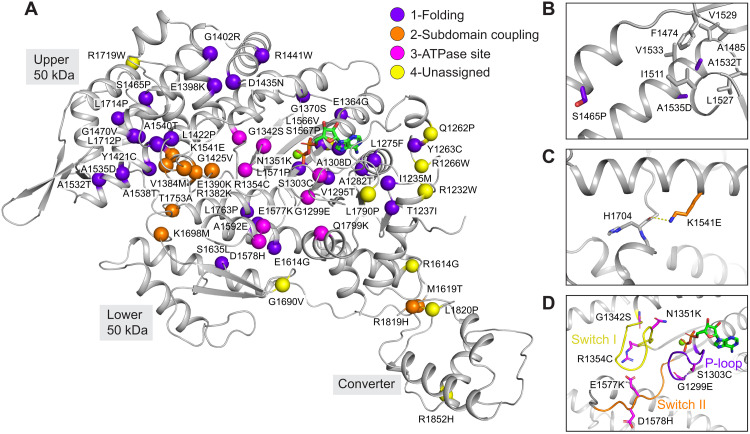
Structural mapping of deafness causing myosin-15 mutations. (**A**) Ribbon diagram of the myosin-15 motor domain with α-carbons at sites of deafness-causing mutations (spheres), colored according to the indicated mechanistic categories. (**B**) Detail view of mutations anticipated to disrupt folding. (**C**) Detail view of mutation anticipated to affect subdomain rearrangements. (**D**) Detail view of mutations in the switch I, switch II, and P-loop regions anticipated to affect ATPase activity.

Within the motor domain of myosin-15, a total of 59 missense mutations targeting 58 residues have been identified in patients with DFNB3 (*8*). Of these 58 residues, 2 are located in disordered regions that were not resolved in our reconstruction: The remaining 56 can be mapped onto the structure (Fig. 2A). Sequence alignment of the mouse and human myosin-15 motor domains shows 54 of the 56 residues are identical and 2 of them are highly similar (fig. S2). Examining the distribution of these deafness-causing mutations leads us to classify them into four groups (Fig. 2A; unless otherwise noted, residues cited correspond to the human protein). Mutations in the first group are found in the core of the motor domain and are likely to disrupt proper folding and thereby completely compromise function. For example, the p.S1465P mutation introduces a proline in the middle of a long helix, and the p.A1532T/p.A1535D mutations substitute alanine with a polar or charged amino acid in the hydrophobic core (Fig. 2B), all of which we anticipate will severely impede folding.

This group additionally includes mouse C1779, the site of the pathogenic p.C1779Y deafness mutation in the *shaker 2* allele, contrary to its originally predicted location at an actin-binding interface (*10*). This residue sits in a hydrophobic core surrounded by mouse I1297, L1275, A1308, and V1777 (fig. S3A), where substitution with a bulky hydrophilic tyrosine would be anticipated to disrupt the proper folding of the transducer. Consistent with this interpretation, recombinantly expressed *shaker 2* (p.C1779Y) motor domain is a soluble aggregate when purified and subjected to size exclusion chromatography (*25*). Furthermore, the human counterpart (A1324) of mouse A1308, which is adjacent to C1779 (fig. S3A), was also found to be substituted with the highly polar residue aspartic acid in a pedigree with profound deafness (*43*), consistent with disruption of the hydrophobic core resulting in misfolding and aggregation.

Mutations in the second group are located in regions mediating interactions between the motor’s subdomains, which are likely to interfere with coordinated rearrangements required to execute the ATPase mechanochemical cycle (*44*). The side chain of the highly conserved K1541 from the upper 50-kDa subdomain forms a hydrogen bond with the backbone of H1704 in the loop, connecting the upper 50-kDa and lower 50-kDa subdomains (Fig. 2C and fig. S2). This interaction likely plays a stabilizing role in the cleft, and the charge inversion p.K1541E mutation is anticipated to disrupt it and hinder cleft closure.

The third group are located in consensus ATPase motifs, including switch I, switch II, and the P-loop. These mutations are expected to have a deleterious impact on the mechanochemical cycle by impeding ATP binding and/or hydrolysis (Fig. 2D and fig. S2). Last, a fourth group of mutations are distributed in solvent exposed loop regions with no obvious pattern (Fig. 2A). We speculate that these residues mediate binding contacts with yet to be identified partner proteins or intramolecular contacts involved in regulating myosin-15’s motor function, which could include an interface between the motor domain and the large 133-kDa N-terminal domain of MYO15A-1; (Fig. 1A) that is required for postnatal stereocilia maintenance (*5*). Our structural mapping analysis suggests that mutations in myosin-15 cause pathology by either interfering with canonical myosin-15 ATPase motor activity or with noncanonical functions such as mediating protein-protein interactions at the stereocilia tips.

### A structurally diversified myosin-15–F-actin interface

**Fig. 3.**
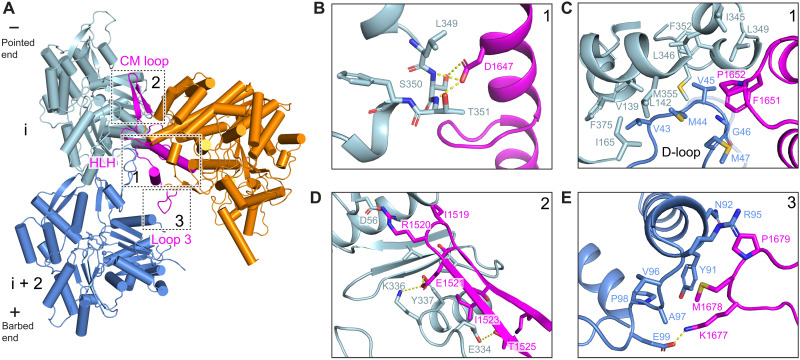
Interactions at the rigor wild-type actomyosin-15 interface. (**A**) Atomic model of the interface between two actin subunits and rigor wild-type myosin-15. Actin subunits i, i + 2, and myosin-15 are colored in light blue, cornflower blue, and dark orange, respectively. The indicated myosin-15 actin interaction motifs are highlighted in magenta, and numbered boxes correspond to detail views in subsequent panels. (**B**) Hydrogen bonding interactions between myosin-15 residue D1647 and actin residues S352 and T353. (**C**) Hydrophobic interactions formed between the D-loop of actin subunit i + 2, subdomains 1 and 3 of actin subunit i, and the HLH motif of myosin-15. (**D**) Interface between the CM loop of myosin-15 and subdomain 3 of actin subunit i. (**E**) Contacts between loop 3 of myosin-15 and subdomain 1 of actin subunit i + 2.

We next undertook a detailed analysis of the myosin-15–F-actin interface to identify specific contacts that could be affected by deafness mutations in both myosin-15 and γ-actin (encoded by the *ACTG1* gene in human), one of the primary actin isoforms expressed in hair cells (fig. S3B), as well as contribute to myosin-15’s F-actin nucleation activity. Similar to other myosins, myosin-15 uses conserved structural elements to interact with F-actin: the Helix-Loop-Helix (HLH) motif, the cardiomyopathy (CM) loop, loop 2, loop 3, and the activation loop (Fig. 3A and fig. S4A). However, residue-level interactions have diversified among myosins to fine-tune the functions of superfamily members (*21*–*23*, *34*, *41*). Myosin-15’s HLH motif (I1641-P1671) mediates the major contact with F-actin through its engagement with two longitudinally adjacent actin subunits (which we here refer to as subunits “i” and “i + 2”) along a protofilament (Fig. 3A). The first helix of the HLH includes myosin-15 residue D1647, the site of the p.D1647G mutation in the *jordan* allele, which forms hydrogen bonds with S350 and T351 on subdomain 1 of subunit i (Fig. 3B). Notably, D1647 is highly conserved among different myosin members, indicating that this interaction is likely broadly important for myosin function (fig. S2). Mutation of this site in myosin-2 resulted in a 10-fold reduction of F-actin binding affinity (*45*), and the F-actin binding affinity of *jordan* mutant myosin-15 is also substantially decreased (*25*).

In the loop portion of the HLH, a pair of hydrophobic residues (F1651 and P1652) inserts into a hydrophobic pocket formed by subdomains 1 and 3 of actin subunit i and the D-loop of subunit i + 2 (Fig. 3C), where L349 of subunit i closely engages with myosin-15 F1651. The γ-actin mutation L349M causes profound deafness in human patients (*46*), and we anticipate replacement of leucine by the bulkier methionine impairs myosin-15 engagement by preventing access to this pocket (Fig. 3C and fig. S3B). In addition, Q1653 forms a hydrogen bond with the backbone of subunit i residue G146 (fig. S4B). The second helix engages with the N-terminal portion of subunit i + 2’s D-loop through a network of electrostatic interactions. Residue K1662 of myosin-15 forms a hydrogen bond with the side chain of D-loop residue Q49, and myosin-15 Y1665 also forms a possible N─H···π bond with this actin residue (fig. S4B).

The activation loop (I1634-G1640) preceding the HLH motif has been suggested to activate myosins through contacts with actin’s negatively charged N terminus (*47*, *48*). K1637 forms a possible salt bridge with subunit i + 2 residue 4E, partially stabilizing actin’s flexible N terminus (fig. S4C), an ionic interaction that is present in both the Mg-ADP and rigor states (fig. S4C). Loop 2 has also been implicated in mediating initial contacts during the weakly bound phase of the myosin mechanochemical cycle before phosphate release (*49*). This region is largely disordered in our structures, suggesting that loop 2 engagement is dispensable for strong binding of myosin-15 to F-actin. Discontinuous density is present for several residues extending from the C-terminal base of loop 2, which orients toward the N terminus of actin and may form weak interactions (fig. S4D). This is in notable contrast to nonmuscle myosin-1b (hereafter “myosin-1”) or nonmuscle myosin-2c (hereafter “myosin-2”), where loop 2 is either partially or fully ordered at the strongly bound interface (*21*, *34*). However, limited loop 2–mediated contacts were also reported in rigor β-cardiac actomyosin (*41*), highlighting a variable role for loop 2 in stabilizing strongly bound actomyosin interfaces across the myosin superfamily.

The CM loop adopts a β-hairpin fold and packs tightly against subdomain 1 of subunit i (Fig. 3, A and D). In contrast to the CM loops of myosin-1, myosin-2, and β-cardiac myosin, which primarily engage in hydrophobic interactions with F-actin (*21*, *34*, *41*), myosin-15’s CM loop-F-actin hydrophobic interface is minimal and is instead dominated by a network of electrostatic interactions. Specifically, the nonconserved residue R1520 at the tip of the CM loop likely mediates a myosin-15–specific salt bridge with actin D56. Similar to myosin-1, the highly conserved residue E1521 mediates electrostatic contacts with actin residues K336 and Y337. The side chain of T1525 also forms a hydrogen bond with actin E334, which is not observed for myosin-1 or myosin-2 (Fig. 3D). Myosin-15 has a relatively short loop 3 that nevertheless mediates substantial contacts with subdomain 1 of subunit i + 2. Compared with myosin-1 and myosin-2, myosin-15’s loop 3 assumes a distinct conformation to form divergent interactions (fig. S4E). Notably, myosin-15 P1679 makes Van der Waals contacts with actin N92 and R95, and M1678 sits in a shallow hydrophobic groove cradled by actin Y91, V96, A97, and P98. This interface is further strengthened by a probable salt bridge between myosin K1677 and actin E99 (Fig. 3E).

Collectively, we observe numerous myosin-15–specific interactions with F-actin, consistent with specialization of this interface for myosin-15 function in stereocilia. At the actomyosin-15 interface, limited loop 2 interactions, unique loop 3 interactions, and primarily polar/electrostatic interactions through the CM loop likely predominate in determining myosin-15’s specific functional properties, as HLH-F-actin interactions are largely conserved in the actomyosin structures reported to date (*21*–*23*, *34*, *41*, *50*). However, as divergent myosins can have similar thermodynamic and kinetic tuning of their mechanochemical cycles, it remains challenging to delineate the roles of individual contacts at the actin-myosin interface, which are likely to modulate the intrinsic structural dynamics of the motor domain to control its function through complex mechanisms (*51*). Future comparative high-resolution structural studies of additional MyTH4-FERM myosins in complex with F-actin, which share substantial motor domain homology with myosin-15 but feature distinct mechanochemical tuning (*4*), may reveal more explicit links between actin-myosin contacts and functional biophysical parameters.

### Rigor myosin-15 evokes structural changes in F-actin while maintaining D-loop flexibility

**Fig. 4.**
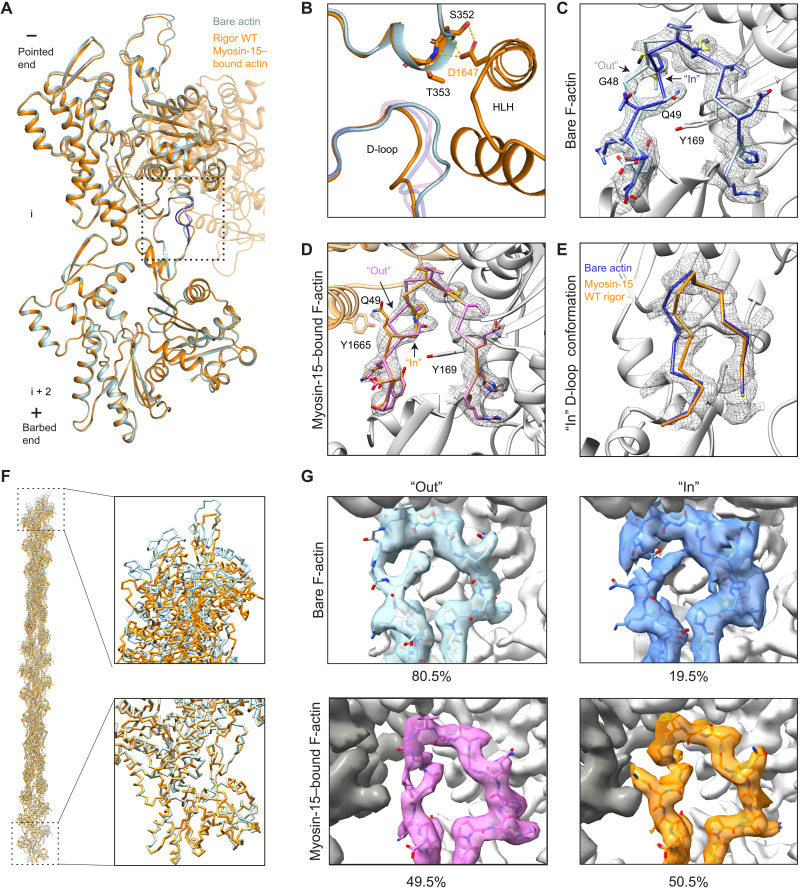
Myosin-15 binding remodels F-actin while maintaining D-loop flexibility. (**A**) Ribbon diagram of two actin subunits in the presence and absence of myosin-15, superimposed on subunit i. Minor D-loop conformations are colored in blue (actin alone) and pink (myosin-15 bound). (**B**) Structural comparison at the interface formed by two longitudinally adjacent actin subunits and the myosin-15 HLH. Bare F-actin and rigor wild-type (WT) actomyosin-15 are colored in light blue and dark orange, respectively. Minor D-loop conformations are colored in blue (actin alone) and pink (myosin-15 bound) and are shown in transparent representation. (**C**) Segmented cryo-EM density of the bare F-actin D-loop rendered at 0.036 RMS, superimposed with stick models of the Out and In D-loop conformations colored in light blue and medium blue, respectively. (**D**) Segmented cryo-EM density of the rigor wild-type myosin-15–bound actin D-loop, superimposed with stick models of the Out and In D-loop conformations colored in dark orange and pink, respectively. (**E**) Backbone representation of the In conformation of the indicated models, superimposed on the segmented cryo-EM density the of myosin-15–bound F-actin D-loop. (**F**) Superposition of actin filament models (31 subunits) from bare F-actin and myosin-15–bound F-actin (see Materials and Methods). The terminal barbed and pointed ends of the filament models are shown in detail views. (**G**) Density maps of two representative three-dimensional (3D) classes of bare F-actin (top) and rigor wild-type myosin-15–bound F-actin (bottom) from focused 3D classification after symmetry expansion showing the D-loop predominantly in the Out (left) and In (right) conformation, respectively.

As rigor myosin-15 potently stimulates F-actin polymerization (*25*), we next compared two longitudinally adjacent actin subunits from our F-actin alone and rigor wild-type actomyosin-15 structures, hypothesizing that myosin-15 could regulate actin polymerization by modulating F-actin conformation. Consistent with previously reported actomyosin structures (*21*, *22*, *34*, *41*), the overall conformation of actin in the presence and absence of myosin-15 is highly similar, with a global root mean square deviation (RMSD) of 0.51 Å (Fig. 4A). Nevertheless, we observe a global compression of the filament along the longitudinal axis (Fig. 4F and movie S1), which is centered around actin rearrangements at the myosin-15–HLH interface (Fig. 4B). Consistently, the helical rise decreases 0.12 Å upon myosin-15 binding, with no substantial alteration of the helical twist (fig. S1C). The short α helix in actin subunit i that forms contacts with D1647 shifts slightly toward the minus end to mediate this compression (Fig. 4B). This is accommodated by rearrangements in the D-loop of subunit i + 2, which is also engaged by the myosin-15 HLH.

Several recent high-resolution cryo-EM studies of F-actin have indicated that the D-loop is structurally dynamic within the context of the filament (*32*, *36*–*38*). At a high threshold (0.036 RMS), our map of bare F-actin features ambiguity surrounding residues G48 and Q49, despite clear density for all other residues in the D-loop (Fig. 4C). However, at a lower threshold (0.025 RMS), our map reveals two continuous paths for the backbone, allowing two distinct conformations of the D-loop to be modeled: An “In” conformation where the main chain of G48 flips in toward the center of the filament and Q49 faces the solvent, and an “Out” conformation where the main chain of G48 is exposed and Q49 is buried in the center of the D-loop (Fig. 4C and fig. S5A). On the basis of the respective strengths of the two density paths, the Out conformation is predominant in the bare F-actin structure, likely stabilized by an interaction between the side chain of Q49 of subunit i + 2 and the side chain of Y169 from the adjacent subunit i (Fig. 4C). Upon rigor myosin-15 binding, the D-loop transitions to primarily being in the In state (Fig. 4, B and D, and fig. S5B) but displaced 2.7 Å outward from the filament axis relative to the bare F-actin conformation (Fig. 4E), enabling an interaction between myosin HLH residue Y1665 and the flipped-out side chain of actin D-loop residue Q49 (Fig. 4D and fig. S5B). Nevertheless, at a lower threshold (0.028 RMS), density resembling the Out conformation in bare actin is clearly visible (Fig. 4D, fig. S5C, and movie S2), suggesting that rigor myosin-15 engages its binding site while maintaining F-actin’s intrinsic structural plasticity in the D-loop. We do not observe specific contacts between the Out conformation and myosin-15, likely explaining why the In state is favored upon myosin-15 binding.

To directly probe the distribution of these D-loop conformational states, we implemented a symmetry expansion (*52*) and focused classification approach (see Materials and Methods and table S2), facilitating separation of D-loop states by avoiding the averaging effects imposed by applying helical symmetry to neighboring protomers, which may adopt different conformations in this region. We first verified that the multiple D-loop conformations observed in the rigor actomyosin-15 reconstruction were not an artifact of averaging a mixture of bare F-actin protomers featuring the Out conformation and myosin-15–bound F-actin protomers in the In conformation. Three-dimensional (3D) classification after symmetry expansion validated that the occupancy of myosin is in 1:1 in stoichiometry with actin when each protomer binding site is treated as an independent particle (see Materials and Methods). The overall resolution of this reconstruction worsened slightly to 3.10 Å; however, two paths are clearly resolved in the D-loop that are identical to what was observed before symmetry expansion (fig. S5D), confirming that rigor myosin-15–bound F-actin indeed features structural dynamics in the D-loop.

Next, we attempted to separate the conformational states of the D-loop in bare F-actin and rigor myosin-15–bound F-actin. Reprocessing the bare F-actin dataset with symmetry expansion yielded a density map at an improved resolution of 2.62 Å (fig. S8). As anticipated, the D-loop continued to display two major conformational states. Further focused 3D classification (*K* = 4) with a tight spherical mask around the D-loop produced well-resolved density in all four classes (Fig. 4G and fig. S5E). Three of the classes (1, 2, and 4), corresponding to 80.5% of the particles, display clear and continuous main chain density for G48 and Q49 in the Out conformation. Moreover, the side chain of Q49 is clearly resolved in classes 2 and 4. However, none of these three classes displays discernable density for the In conformation, even at low (0.015 RMS) thresholds, indicating that the D-loop in these classes predominantly adopts the Out conformation. Class 3, corresponding to 19.5% of the particles, instead showed discontinuous density for the main chain spanning G48 and Q49 at a high threshold (0.018 RMS). At a lower threshold (0.015 RMS), density paths for both the Out and In conformations emerge. In contrast to the predominant Out conformation in classes 1, 2, and 4, the density for the In conformation is moderately stronger than the Out conformation in class 3, implying that the D-loop still represents a mixture of states but preferably assumes the In conformation (Fig. 4G). This analysis further supports a model in which the D-loop of bare F-actin dynamically samples two conformations, with the Out conformation being predominant.

Applying the same focused 3D classification procedure (*K* = 4) to the rigor myosin-15–bound F-actin dataset produced three classes with well-resolved D-loops and one empty junk class. Classes 1 and 2, corresponding to 50.5% of the particles, exhibited clear and continuous density for main chain G48 and Q49 solely in the In conformation, with the flipped side chain of Q49 clearly resolved in class 2 (Fig. 4G and fig. S5F). In contrast, well-resolved density for the Out conformation is present in class 4, corresponding to 49.5% of the particles, with weak density corresponding to the In conformation (Fig. 4G). Therefore, we conclude that rigor-state myosin-15 binding maintains flexibility in actin’s D-loop but shifts the distribution toward the In conformation.

### The myosin-15 *jordan* deafness mutant locks the D-loop in the In conformation

**Fig. 5.**
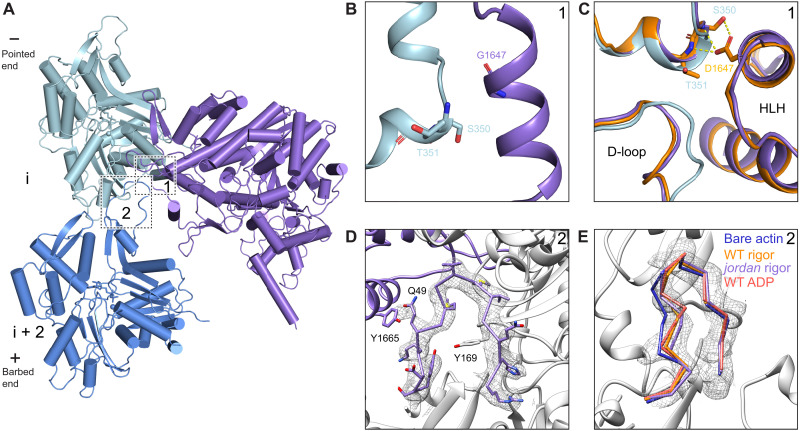
Binding by rigor *jordan* mutant myosin-15 locks the actin D-loop. (**A**) Atomic model of the interface between two actin subunits and rigor *jordan* myosin-15. Actin subunits i, i + 2, and myosin-15 are colored in light blue, cornflower blue, and medium purple, respectively. Numbered boxes correspond to detail views in subsequent panels. (**B**) Actomyosin interface at the site of p.D1647G mutation. (**C**) Structural comparison of bare F-actin (light blue), rigor wild-type (dark orange), and *jordan* mutant (medium purple) myosin-15–bound F-actin at the interface formed by two longitudinally adjacent actin subunits and the myosin HLH. (**D**) Segmented cryo-EM density and atomic model of the rigor *jordan* mutant actomyosin-15 D-loop. (**E**) Backbone diagram of the three In D-loop conformation models superimposed on segmented cryo-EM density of the rigor *jordan* mutant actomyosin-15 D-loop.

To gain insight into structural mechanisms underlying the *jordan* mutant’s suppression of actin assembly and disruption of stereocilia growth (*25*), we next compared the rigor structures of wild-type and *jordan* mutant actomyosin-15. Despite similar numbers of segments being incorporated into the final wild-type (142,635) and *jordan* mutant (91,340) reconstructions, the overall resolution of the *jordan* mutant reconstruction was notably worse, and its radial decay in resolution more severe (fig. S1), suggesting that the mutant actin-myosin interface may feature increased flexibility. Nevertheless, the overall conformations of the motor domains are essentially indistinguishable, arguing that the *jordan* mutation does not grossly compromise myosin-15’s structure (fig. S6A). The D1647G substitution abolishes the contacts formed between D1647 and actin residues S350 and T351 (Fig. 5, A and B) without disrupting the conformation and positioning of the HLH or discernibly perturbing any other contacts at the myosin-15–F-actin interface (fig. S6, B to E). We next examined the structures for global differences in actin conformation, hypothesizing that these could underlie the differential effects upon actin polymerization. Morphing the density maps reveals minimal changes, and the refined helical parameters of the reconstructions are nearly identical (fig. S1C and movie S3), indicating that the *jordan* mutation does not substantially affect the global structural rearrangements in F-actin evoked by myosin-15. Consistently, the short α-helix comprising S350 and T351 of subunit i still undergoes a shift indistinguishable from that observed in the wild-type reconstruction (Fig. 5C), indicating that this conformational transition is not solely evoked by contacts formed with D1647 and is instead likely an allosteric effect driven by overall engagement of the myosin-15–F-actin interface. Collectively, these data suggest that the *jordan* mutation also does not grossly disrupt the actin–myosin-15 interface or myosin-15’s overall conformational remodeling of F-actin.

We thus turned to an alternative hypothesis, wherein the *jordan* mutant disrupts actin structural plasticity rather than global actin conformation. The D-loop is clearly resolved in the *jordan* mutant reconstruction, and the backbone and side chain of each residue can be fully fit into the density, adopting an In conformation indistinguishable from the wild-type reconstruction (Fig. 5, C and D). Density for the Out conformation, on the other hand, is completely absent, demonstrating that the *jordan* mutant locks the D-loop in the In conformation and eliminates its flexibility (movie S4). Consistent with the helically averaged map, symmetry expansion and focused classification (*K* = 4) failed to recover any classes with Out D-loop density. Instead, 30.6% of the particles partitioned into a single interpretable class with In D-loop density, with the remaining sorted into three junk classes with poor quality density (fig. S9B). Thus, rather than eliciting specific structural rearrangements adjacent to the site of the p.D1647 lesion, breaking the myosin-15–F-actin contacts mediated by D1647 instead unexpectedly restricts the structural plasticity of the D-loop, potentially through long-range allosteric effects. While we cannot rule out the possibility that the *jordan* mutation elicits additional perturbations of myosin-15 or F-actin structure during other steps of the myosin mechanochemical cycle, wild-type myosin-15 most potently stimulates F-actin assembly in the absence of nucleotide, arguing that the *jordan* mutant’s defect likely occurs in a strongly bound state (*25*). As restricted D-loop flexibility is the only distinguishable difference between the two rigor structures, we propose that this dysregulation of actin structural plasticity contributes to inhibition of actin assembly by the *jordan* mutant (*25*). Our structures furthermore support a key role for actin residue G48 in conferring D-loop flexibility mediating F-actin assembly. In γ-actin, the p.G48R substitution (fig. S3B) has been documented to cause progressive hearing loss in patients (*53*). While D-loop flexibility at this position is likely a general feature of mammalian actin isoforms (*54*, *55*), our data suggest that it is specifically required for efficient actin polymerization in stereocilia.

### The Mg-ADP state of myosin-15 stabilizes the D-loop to restrict actin nucleation

**Fig. 6.**
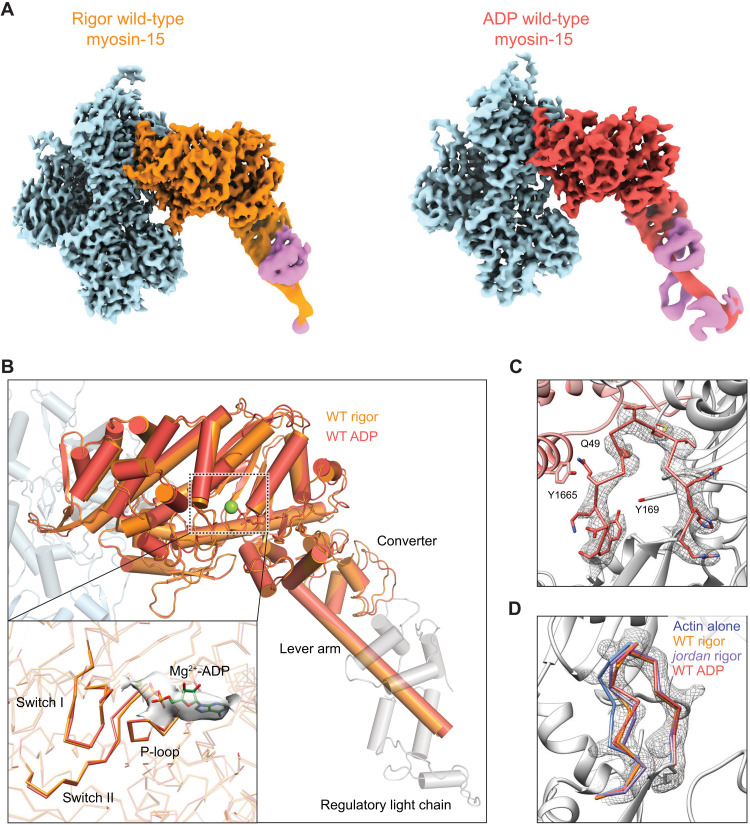
ADP-bound myosin-15 undergoes minimal rearrangements and locks the D-loop in the In conformation. (**A**) Cryo-EM maps of indicated actomyosin-15 complexes derived from reprocessing with symmetry expansion and recentering on myosin-15’s residue S1245. (**B**) Superposition of rigor and ADP wild-type actomyosin-15 atomic models. Actins from the rigor atomic model are shown in shades of transparent blue. Inset: Detail view of structural elements in the nucleotide-binding pocket before and after ADP release. (**C**) Cryo-EM map and corresponding atomic model of actin’s D-loop in ADP wild-type actomyosin-15. (**D**) Backbone positioning of In D-loop conformation in the indicated models, superimposed on the segmented cryo-EM map of the ADP wild-type actomyosin-15 D-loop.

The ability of myosin-15 to nucleate F-actin is reduced in the presence of ATP (*25*), leading us to hypothesize that other states in the motor’s mechanochemical cycle could have distinct effects on actin polymerization by differentially regulating F-actin conformation, analogous to myosin-6’s nucleotide state–dependent modulation of F-actin conformation through its contact with the D-loop (*22*). We next undertook a detailed comparison of the rigor and Mg-ADP–bound actomyosin-15 wild-type structures. The overall conformation of the myosin-15 motor domain is highly similar in both reconstructions, with a global RMSD of 0.462 Å for 493 aligned C_α_ atoms (Fig. 6B), indicating an absence of major subdomain rearrangements during ADP release (fig. S7A). Clear density corresponding to ADP is present in the Mg-ADP–state cryo-EM map (Fig. 6B), arguing that the observed lack of rearrangements is not due to insufficient nucleotide binding by the motor. Moreover, unlike myosin-1 (*21*), where two ADP-bound states have been reported, 3D classification produced no evidence of multiple ADP states in myosin-15 (see Materials and Methods).

Myosin-1, myosin-5, and myosin-6 all feature a minor lever-arm swing accompanying ADP release (fig. S11), which has been postulated to mediate the known force-sensitivity of this step in their mechanochemical cycles (*21*–*24*). In our helically averaged structures, the short region of the myosin-15 lever arm that was resolved does not undergo a substantial transition (fig. S7A). To more rigorously probe whether even a minor transition accompanying ADP release occurs, which might only be discernable in more distal regions of the lever arm, we once again implemented a symmetry expansion approach for the wild-type rigor and ADP-state actomyosin-15 complexes, this time recentering the particles on the myosin motor domain to facilitate alignments capturing the distal converter and lever arm regions (see Materials and Methods and table S2). This produced reconstructions with substantially improved density in the nucleotide binding pocket and lever arm region. Both structures showed clear secondary structural density of the lever arm till the end of the first IQ motif and the C-terminal lobe of the associated light chain (Fig. 6A and fig. S8). Biochemical analysis demonstrated that the first myosin-15 IQ motif binds the regulatory light chain (RLC) rather than the essential light chain (ELC), as is canonically found in myosin-2 (*4*, *56*), the functional implications of which remain unclear. Our density map displays all four α helices corresponding to the C-terminal lobe and only two discernable α helices at the N-terminal lobe (Fig. 6A and figs. S8 and S10). Superposition of the RLC model from chicken skeletal muscle myosin and that of myosin-15 reveals a similar conformation and pose of the C-terminal lobe, while the relative orientation of the N-terminal lobes diverges (fig. S10). The conformation of the myosin-15 RLC is also distinct from the skeletal muscle myosin ELC (fig. S10), as well as the myosin-5 RLC (*23*), and appears unique among myosins that have thus far been structurally characterized. Whether this divergence plays a role in tuning myosin-15’s mechanochemical cycle will be an important topic for future studies (fig. S10). Superimposing the rigor and ADP models built from these maps (see Materials and Methods) clearly reveals the absence of a lever arm swing upon ADP release, unlike myosin-1, myosin-5, or myosin 6 (Fig. 6B and fig. S11). An essentially complete lack of coupling between ADP release and lever arm repositioning strongly suggests that this step of the mechanochemical cycle is unlikely to be meaningfully mechanically gated in myosin-15.

We next examined the nucleotide binding pockets of these two states, which were well resolved at the secondary structure level by the symmetry expansion and myosin-recentering procedure. Unexpectedly, other than the clear presence of nucleotide density in the ADP state and its absence in the rigor state, we observe no discernable rearrangements in the backbone positioning of the nucleotide binding loops upon ADP release (Fig. 6B), unlike myosin-1, myosin-5, or myosin-6 (*21*–*23*). While the limited resolution in this region of the maps does not rule out more subtle side-chain level repositioning, our data imply that any major rearrangements in myosin-15’s nucleotide binding pocket accompanying ATP hydrolysis must occur concomitant with phosphate release, before strong binding on F-actin. This rationalizes the reported modest coupling between myosin-15’s ADP binding and actin binding (*16*), and it furthermore suggests that myosin-15 uses a distinct ADP release mechanism from other structurally characterized actomyosin complexes (*21*–*23*).

We next compared the conformation of F-actin between the rigor and Mg-ADP structures. Consistent with the lack of conformational rearrangements in the motor domain, ADP release by myosin-15 evokes minimal global conformational changes in F-actin (movie S5); correspondingly, the helical rise of the two reconstructions is nearly identical (fig. S1C). However, density for the D-loop is clearly resolved solely in the In conformation, positioned as we observed in both the wild-type and *jordan* mutant rigor reconstructions. Similar to the *jordan* mutant actomyosin-15 reconstruction, and unlike the rigor wild type, density for the Out conformation is completely absent (Fig. 6, C and D, and movie S6). Consistently, as was the case with the *jordan* mutant, symmetry expansion and focused classification (*K* = 4) only recovered a single interpretable class, clearly featuring the In conformation (fig. S9A). Because rigor *jordan* myosin-15 suppresses both D-loop dynamics and actin polymerization, we hypothesized ADP binding would also correspondingly reduce the actin-polymerization stimulating activity of wild-type myosin-15 by also stabilizing the D-loop in the In conformation.

**Fig. 7.**
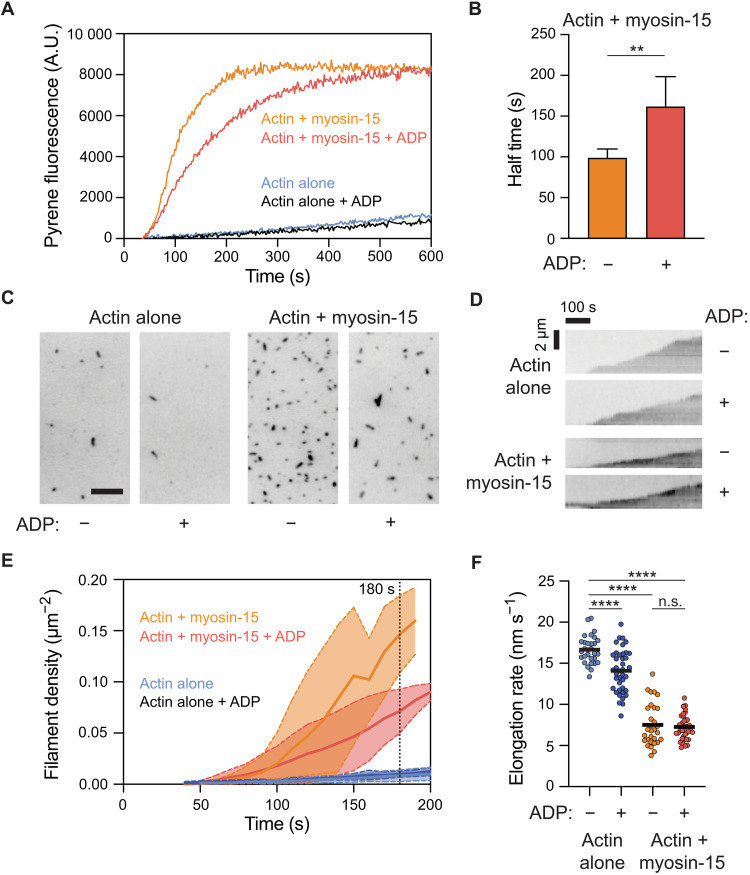
ADP bound myosin-15 blunts F-actin nucleation. (**A**) Representative pyrene actin polymerization assays in the presence and absence of myosin-15 and ADP. Actin, 2 μM; myosin-15, 1 μM; Mg-ADP, 100 μM. A.U., arbitrary units. (**B**) Quantification of the time to half-maximal pyrene signal saturation in the presence of myosin-15 with or without ADP. Error bars represent SD. *n* = 5 from three independent protein purifications; **P = 0.008, Mann-Whitney *U* test. (**C**) Snapshots after 180 s of actin polymerization from TIRF movies recorded in the presence and absence of myosin-15 and ADP. Scale bar, 10 μm. (**D**) Quantification of F-actin density, indicative of nucleation rate, from TIRF movies. Solid lines and dashed lines/shading represent means ± SD at each time point; *n* = 3. (**E**) Representative kymographs of elongating individual actin filaments from TIRF movies. (**F**) Quantification of filament elongation rates from TIRF movies. *n* = 3 independent replicates. Bars represent mean; ****P < 0.0001, one-way analysis of variance. n.s., not significant.

Consistent with this prediction, we observe a slower rate of actin assembly when saturating Mg-ADP is included along with myosin-15 in pyrene actin polymerization assays versus nucleotide-free conditions (Fig. 7A), with an approximate doubling of the half-time to reach steady state (Fig. 7B). Inclusion of Mg-ADP did not substantially affect the assembly rate of actin alone (Fig. 7A), suggesting that ADP down-regulates the polymerization-stimulating activity of myosin-15 rather than perturbing F-actin formation. We next sought to determine whether ADP binding by myosin-15 affected the nucleation or elongation phases of actin polymerization by directly visualizing filament dynamics with total internal reflection fluorescence (TIRF) microscopy (Fig. 7C). Relative to actin alone, we observed a notable increase in the density of filaments per unit area in the presence of myosin-15, which was blunted by the inclusion of saturating Mg-ADP (Fig. 7, C and D), suggesting a specific effect on actin nucleation. When the growth of individual filament plus ends was monitored (Fig. 7E), both nucleotide-free and Mg-ADP myosin-15 significantly slowed elongation to ~7.5 nm/s, relative to ~16 nm/s for actin alone (Fig. 6I). This rate was indistinguishable from that observed in the presence of the rigor *jordan* mutant, presented in our companion study (*25*). As a further control, the addition of ADP only modestly affected the elongation rate of actin alone (Fig. 7F). Myosin-15’s opposing effects of stimulating nucleation while reducing elongation strongly suggest that the overall increased F-actin assembly rate is dominated by myosin-15’s effect on stimulating nucleation, as previously reported for muscle myosin S1 (*27*, *28*), in a manner that is specifically regulated by myosin-15 nucleotide state.

## DISCUSSION

Here, we establish structural mechanisms underpinning myosin-15’s function in constructing the actin core of stereocilia, a process that is critical for hearing. Our actomyosin-15 cryo-EM structures, the first myosin-15 motor domain structures, to our knowledge, of any kind, assign the likely mechanistic deficiencies associated with mutations causing autosomal recessive deafness DFNB3 and provide a framework for interpreting yet to be identified clinical variants (Fig. 2). Many DFNB3 mutations are intuitively anticipated to affect the motor’s mechanochemical cycle. However, our structure also highlights surface substitutions that are not readily interpreted through this lens, suggesting that additional functions of the myosin-15 motor domain important for hearing remain to be identified, such as serving as a protein-protein interaction hub. Our studies additionally reveal how myosin-15 modulates actin’s structural landscape to enhance F-actin nucleation in a manner controlled by the motor’s nucleotide state. We speculate this activity could be harnessed to provide fine-tuned control of F-actin assembly at stereocilia tips, facilitating the precise control of stereocilia height required for sound detection.

**Fig. 8.**
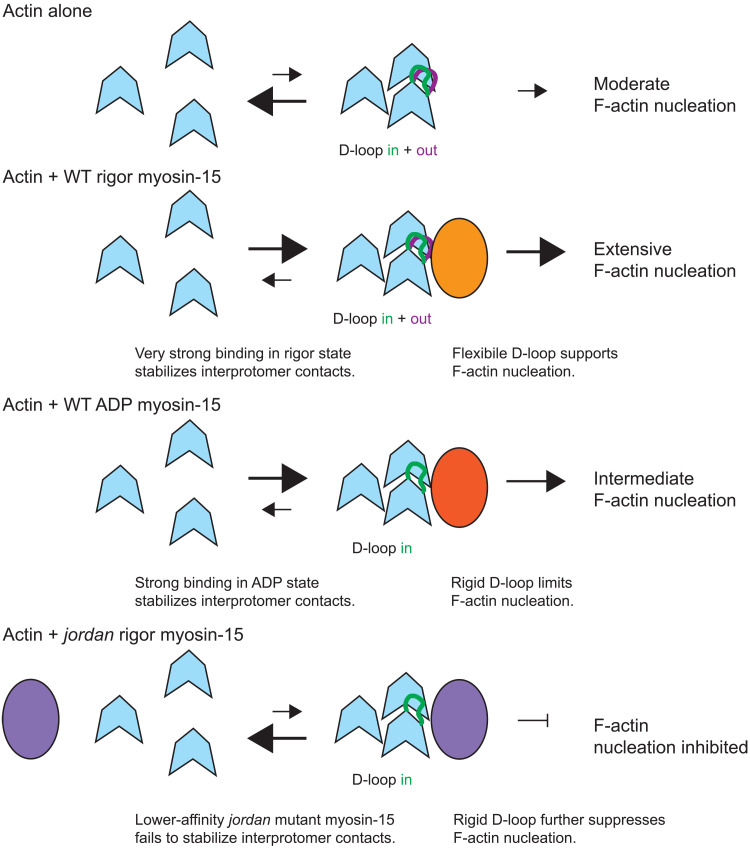
Schematic model for regulation of actin polymerization by myosin-15. We propose myosin-15 enhances F-actin formation by stimulating nucleation, both by stabilizing actin-actin contacts and modulating D-loop structural plasticity. Myosin-15 nucleotide state facilitates tuning of nucleation activity, which could be harnessed for stereocilium height control. The *jordan* mutation disrupts F-actin regulation, thereby inhibiting polymerization.

Our cryo-EM reconstructions and associated functional data reveal subtle structural plasticity in the actin D-loop that likely mediates regulated F-actin assembly by myosin-15. While substantial D-loop rearrangements accompanying the soluble G-actin to polymerized F-actin conformational transition are well established (*33*), the extent and functional role of D-loop rearrangements within F-actin remain controversial. Substantially different “open” and “closed” conformations have been reported in cryo-EM reconstructions of F-actin in the presence of stabilizing drugs and actin nucleotide-state analogs (*36*, *37*) leading to speculation that an open-to-closed transition is associated with nucleotide hydrolysis and phosphate release concomitant with F-actin polymerization. However, others have reported minimal rearrangements when comparing highly similar conditions, with the D-loop constitutively adopting a closed conformation in F-actin (*32*, *38*). The data we present here reveal the closed D-loop conformation in ADP F-actin to be a mixture of In and Out states whose occupancy can be manipulated by myosin-15 engagement. Wild-type rigor myosin-15 binding retains both the In and Out D-loop conformations and strongly nucleates actin polymerization, while ADP-bound wild-type myosin-15 and rigor *jordan* mutant-15 both lock the D-loop in the In state, with corresponding moderate and notable reductions, respectively, in nucleation activity (Fig. 8). Collectively, these data suggest that the coexistence of the In and Out D-loop conformations promotes polymerization relative to locking the D-loop in the In conformation, a model that is consistent with a recent report demonstrating that constraining D-loop flexibility with short-distance cross-linkers was refractory to actin polymerization and stimulated F-actin disassembly (*32*). Previous studies have further shown that muscle myosin S1 can rescue polymerization of actin featuring a proteolytically nicked D-loop (*57*), consistent with a role for myosin in modulating D-loop dynamics necessary for actin polymerization.

Nevertheless, additional mechanisms beyond D-loop plasticity are likely required to explain wild-type myosin-15’s stimulation of actin polymerization and its notable disruption by the *jordan* deafness mutation. While actin polymerization by muscle myosin S1 has been subjected to extensive biochemical scrutiny, the exact mechanism remains controversial. Chaussepied and colleagues (*27*) have suggested that each S1 engages a single G-actin, which then stimulates polymerization through a standard nucleation-elongation mechanism. Carlier and coworkers (*28*), on the other hand, have suggested that each S1 engages two G-actins, forming trimers with an F-actin-like actin-actin longitudinal bond that coalesce into filaments through a mechanism distinct from standard polymerization. While our data do not directly discriminate between these mechanisms, our observation that the *jordan* mutation is localized to the HLH interface, which bridges the contact between two longitudinally adjacent protomers, supports a model in which high-affinity myosin-15 binding promotes longitudinal interactions to stimulate polymerization (Fig. 8). A similar mechanism has been proposed for the Spire family of actin nucleators, and the Spire homolog JMY has been detected in stereocilia by mass spectrometry (*58*). However, to our knowledge, no hearing phenotypes have been associated with JMY mutations (*58*), suggesting that JMY is unlikely to make a major contribution to actin assembly in stereocilia.

The modestly lower affinity of ADP–myosin-15 and notably lower affinity of the *jordan* mutant for F-actin, relative to the rigor wild type (*25*), thus also likely makes a major contribution in determining their potency for stimulating F-actin formation, which may, in fact, be dominant over D-loop dynamics. This defect occurring at the level of nuclei formation, rather than elongation, is consistent with our observation that F-actin elongation rates are indistinguishable in the presence of ADP-bound or rigor wild-type myosin-15 (Fig. 7, E and F), as well as the rigor *jordan* mutant (*25*), all of which slow actin polymerization relative to G-actin alone. Regardless, diminished affinity of the *jordan* mutant cannot be the sole explanation for its nucleation defect, as the presence of this myosin, which nevertheless binds F-actin with measurable affinity in an almost identical pose to the wild type, actually initially suppresses the rate of F-actin formation relative to G-actin alone (*25*). The most parsimonious explanation for this effect of the *jordan* mutation is through the suppression of D-loop flexibility as highlighted above, emphasizing the contributions of both myosin-15 bridging actin monomers and the wild type’s maintenance of actin conformational plasticity to stimulating nucleation and the overall accelerated actin polymerization we observe in its presence (Fig. 8).

A limitation of our study is that we only examined myosin-15 in complex with ADP-bound F-actin, and myosin-15 may exert additional structural effects on other actin nucleotide states relevant during polymerization beyond those we describe here. In a cryo-EM structural study contemporaneous with this report, myosin-5 binding was found to overrule actin-nucleotide state–dependent rearrangements in the D-loop, constitutively locking it in a closed conformation, which was not further analyzed for the presence of the more subtle In and Out states reported here (*23*). Previous work has furthermore suggested that skeletal myosin S1 also binds and stimulates slow polymerization of ADP actin, supporting a model in which myosin nucleates actin polymerization through the nonstandard mechanism described above proposed by Carlier and coworkers (*59*). In future studies, it will be important to visualize myosin-15 bound to multiple nucleotide states of F-actin, as well as assess the actin nucleating activity of other unconventional myosins (including myosin-5) to determine whether modulating D-loop flexibility is a general feature of myosin-stimulated F-actin nucleation.

Our studies define a biophysical and structural framework for tunable stimulation of actin polymerization by myosin-15 that could be modulated by the local biochemical environment at the tips of stereocilia, the major site of actin polymerization. The cell biological logic of requiring a myosin motor to nucleate actin in this compartment is not immediately intuitive, as stereocilia tips are rich with F-actin barbed (plus) ends, which, in principle, should be available for elongation. Nevertheless, the phenotype of the *jordan* mouse strongly suggests that myosin-15–induced nucleation is required for stereocilia elongation (*25*). Recent evidence reveals the requirement for de-capping and/or severing of preexisting stereocilia actin filaments for their subsequent elongation (*60*). Short myosin-15–nucleated filaments could then anneal with the ends of preexisting filaments (*61*), analogous to the coalescence mechanism proposed by Carlier and coworkers (*28*) for myosin-stimulated actin polymerization by muscle S1 in vitro. Future high-resolution cryo–electron tomography studies, which are increasingly becoming feasible (*62*), will be important for establishing the precise cytoskeletal network architecture associated with assembling filaments at stereocilia tips.

We speculate that hair cells harness the differential efficiency of actin nucleation by myosin-15 in distinct nucleotide states as a regulatable mechanistic component of stereocilia height control. In our companion manuscript, we report that ATP markedly reduces the actin nucleation activity of myosin-15, to a much greater degree than we report here for ADP (*25*). It is thus feasible that the ADP-bound state, which substantially accelerates actin polymerization, albeit less than the rigor state, is sufficient for supporting myosin-15’s direct contribution to actin nucleation in the tip compartment. Within this framework, control over myosin-15’s actin nucleation could be exerted by the availability of ATP versus ADP (*25*), which has previously been reported to be dynamically maintained by creatine kinase in stereocilia during mechanotransduction and adaptation (*63*) in a manner that may vary in different species and hair cell subtypes (*58*). Our structural data furthermore suggest that ADP release is unlikely to be mechanically gated in myosin-15, which could facilitate the motor populating a nucleotide-free state in the cell to boost its nucleation activity in certain ATP/ADP concentration regimes. In future studies, it will be important to determine the dynamic availability of ATP and ADP in the tip compartment of developing stereocilia and how myosin-15’s direct effects on actin polymerization are coordinated with the WHRN-EPS8-GPSM2-GNAI3 elongation network that it transports. Other environmental conditions known to affect stereocilium integrity, such as oxidative stress (*64*), could also potentially affect actin nucleation by myosin-15, both by dysregulation of nucleotide availability and by direct modification of the myosin-15 protein, disrupting the actin-myosin interface in a manner conceptually analogous to the *jordan* mutation. Last, our observation that the *jordan* mutation occurs at a conserved position suggests that other unconventional myosins could also feasibly stimulate actin polymerization in stereocilia and in diverse physiological contexts beyond hair cells. The mechanistic framework that we establish here will broadly facilitate investigating the functional role of unconventional myosins in actin assembly dynamics.

## MATERIALS AND METHODS

### Expression and purification of myosin-15

Baculoviruses encoding either the wild-type or *jordan* variant of the mouse myosin-15 motor domain (NP_874357.2, amino acids 1 to 743) truncated after the second IQ domain, and including a C-terminal enhanced green fluorescent protein and FLAG moiety, were produced as described (*25*). *Sf*9 cells were seeded at 2 × 10^6^ cells/ml in ESF-921 (Expression Systems) and infected simultaneously with myosin-15 baculovirus at a multiplicity of infection (MOI) of 5. Additional dual-promoter baculovirus expressing bovine smooth muscle ELC (MYL6) and chicken RLC (MYL12B; MOI = 5), in addition to mouse UNC45B and HSP90AA1 (MOI = 5) were included (*4*). Cells were harvested 48 to 72 hours after infection and flash-frozen in liquid nitrogen.

Myosin-15 motor domains were purified as described (*25*). Briefly, cells were homogenized in extraction buffer: 10 mM Mops, 500 mM NaCl, 1 mM EGTA, 10 mM MgCl_2_, 2 mM ATP, 0.2 mM phenylmethylsulfonyl fluoride (PMSF), 0.1 mM dithiothreitol (DTT), 1 mM NaN_3_, leupeptin (2 μg/ml), and 1× protease inhibitor cocktail (Halt EDTA-free; Thermo Fisher Scientific) (pH 7.2). Lysates were clarified at 48,000*g* for 30 min and incubated with FLAG M2 affinity resin (Sigma-Aldrich) for 3 hours at 4°C. The FLAG resin was then washed in high-salt buffer [10 mM Mops, 500 mM NaCl, 1 mM EGTA, 5 mM MgCl_2_, 1 mM ATP, 0.1 mM PMSF, 0.1 mM DTT, 1 mM NaN_3_, and leupeptin (2 μg/ml) (pH 7.2)], followed by low-salt buffer [10 mM Mops, 60 mM NaCl, 1 mM EGTA, 0.1 mM PMSF, 0.1 mM DTT, 1 mM NaN_3_, and leupeptin (2 μg/ml) (pH 7.0)]. Myosin-15 protein was subsequently eluted with 3x FLAG peptide (0.2 mg/ml; American Peptide, CA) in low-salt buffer. Eluted myosin-15 motor domain was then purified by anion exchange chromatography (5/50 MonoQ GL, Cytiva). After injecting the sample, the column was washed with 10 mM Mops, 100 mM NaCl, 1 mM EGTA, 0.1 mM PMSF, and 1 mM DTT (pH 7.0) and then eluted with a linear gradient to 1 M NaCl. Fractions eluting at ∼31 mS/cm were concentrated [10,000 molecular weight cutoff (MWCO)] and further purified by size exclusion chromatography (Superdex 200, Cytiva) with isocratic elution in 10 mM Mops, 100 mM KCl, 0.1 mM EGTA, 1 mm NaN_3_, 0.1 mM PMSF, 1 mM DTT, and leupeptin (1 μg/ml) (pH 7.0). The myosin-15:ELC:RLC complex (1:1:1) eluted as a single peak and was concentrated (10,000 MWCO) before determining concentration (A280; ε = 88,020 M^−1^ cm^−2^). Purity was confirmed by SDS–polyacrylamide gel electrophoresis (4 to 20% TGX; Bio-Rad).

### Actin purification

Chicken skeletal muscle actin was purified as previously described (*65*) and stored in G-Mg buffer: 2 mM tris-HCl (pH 8.0), 0.5 mM DTT, 0.2 mM MgCl_2_, and 0.01% NaN_3_ at 4°C. F-actin was prepared by mixing 5 μM monomeric actin with KMEI buffer [50 mM KCl, 1 mM MgCl_2_, 1 mM EGTA, and 10 mM imidazole (pH 7.0)] supplemented with 0.01% NP-40 substitute (Roche). The mixture was incubated at room temperature for 1 hour to stimulate polymerization, followed by 4°C overnight. NP-40 is included for cryo-EM specimen preparation purposes, as we have found it promotes the consistent formation of evenly distributed thin ice films across the grid for many cytoskeletal filament samples.

### Grid preparation

For the F-actin alone sample, 3 μl of F-actin diluted to 0.6 μM in KMEI + NP-40 was applied to a glow-discharged C-flat 1.2/1.3 holey carbon Au 300-mesh grid (Electron Microscopy Sciences) in a Leica EM GP plunge freezer operating at 25°C. After 60 s of incubation, the grid was blotted from the back with Whatman no. 5 filter paper for 4 s and flash-frozen in liquid ethane. To prepare the rigor wild-type and *jordan* mutant myosin-15–bound F-actin samples, apyrase (10 U/ml) was added to 6 μM myosin-15 and incubated on ice for 20 min to remove nucleotide. Sixty seconds after applying 3 μl of 0.6 μM F-actin to a glow discharged grid, 3 μl of myosin-15 was added and incubated for 60 s. After that, 3 μl of the mixture solution was removed, and another 3 μl of myosin-15 was applied. After an additional 60 s of incubation, 3 μl of the mixture solution was removed, and the grid was blotted for 4 s and flash-frozen in liquid ethane. The ADP-Mg–state wild-type myosin-15–bound F-actin specimen was prepared identically, except that wild-type myosin-15 was incubated with 5 mM ADP-Mg for 20 min before grid preparation.

### Cryo-EM data collection

Data were collected on a FEI Titan Krios operating at 300 kV equipped with a Gatan K2-summit detector using super-resolution mode. Exposures were targeted using stage translations with a single exposure per hole using the SerialEM software suite (*66*), and movies were recorded at a nominal magnification of ×29,000, corresponding to a calibrated pixel size of 1.03 Å at the specimen level (super-resolution pixel size of 0.515 Å per pixel). Each exposure was fractionated across 40 frames with a total electron dose of 60 *e*^−^/Å^2^ (1.5 *e*^−^/Å^2^ per frame) and a total exposure time of 10 s, with defocus values ranging from −1.5- to −3.5-μm underfocus.

### Cryo-EM image processing

Image processing was carried out using the IHRSR protocol as implemented in the RELION 3.0 pipeline (*39*, *40*). Unless otherwise noted, all steps were carried out in RELION. Movie stacks were motion-corrected, dose-weighted, and summed with 2 × 2 binning (to a 1.03-Å pixel size) using the MotionCor2 algorithm (*67*) with 5 × 5 patches as implemented in RELION. Contrast transfer function (CTF) estimation was performed with CTFFIND4 (*68*). Filaments were autopicked and split into overlapping segments with a step size of 81 Å, corresponding to three actin protomers, then extracted in a box size of 512 pixels, and subjected to 2D classification. Segments contributing to featureful class averages were selected and subjected to 3D classification with four classes, initialized with helical parameters of 27-Å rise and −166.7° twist. The cryo-EM maps of F-actin alone (EMD-7115) and myosin VI–bound F-actin (EMD-7116) were low pass filtered to 35 Å to serve as the initial models for F-actin alone and myosin-15–decorated F-actin datasets, respectively. The best 3D class from each dataset was selected and low-pass–filtered to 35 Å to serve as the initial reference for subsequent 3D autorefinement with helical symmetry (averaging across three asymmetric actin protomers). Segments contributing to all the 3D classes, which did not substantially vary in features, were included for 3D autorefinement. After 3D autorefinement, postprocessing was performed for each dataset using a 3D mask with a Z length of 50% of the box size, resulting in maps with 4.23 Å (actin alone), 4.23 Å (rigor wild-type myosin-15), 6.16 Å (rigor *jordan* myosin-15), and 5.56 Å (ADP wild-type myosin-15) resolution based on the Fourier shell correlation (FSC) 0.143 criterion.

To further improve the resolution, iterative CTF refinement, Bayesian polishing, and 3D autorefinement was carried out by adapting a recently described procedure (*35*). Briefly, CTF refinement was initially performed without beam-tilt estimation, followed by another round of 3D autorefinement using the converged map from the last refinement as the initial reference, low-pass–filtered to 35 Å. Subsequent postprocessing was performed using a 3D mask with a Z length of 30% of the box size and then a second round of CTF refinement with beam-tilt estimation, Bayesian polishing, and 3D autorefinement using the converged map from the previous refinement low-pass–filtered to 35 Å as the initial reference. A final masked refinement was performed for the rigor wild-type reconstruction using the 30% Z length mask, which modestly improved the resolution: Masked refinement did not improve either of the other actomyosin reconstructions. Final postprocessing for each dataset was performed with a 30% Z length mask, leading to final resolution assessments of 2.82 Å (F-actin alone), 2.83 Å (rigor wild-type myosin-15), 3.76 Å (rigor *jordan* mutant myosin-15), and 3.63 Å (ADP wild-type myosin-15) by the FSC 0.143 criterion (fig. S1). Local resolution estimation and filtering was performed using RELION 3.0. Data acquisition and processing details are listed in table S1, and final refined helical parameters are listed in fig. S1D.

To improve the occupancy of myosin-15 bound to F-actin, an approach combining symmetry expansion and focused 3D classification was applied. The particles from the final helical refinement with three asymmetric actin protomers were symmetry expanded by generating three copies of each particle, modulating the alignment parameters by the helical rise and twist of a single actin protomer (*52*). This leads to tripling of the particle number, with each particle containing only one asymmetric actin protomer. Masks covering the central three actin and one myosin subunits for the actomyosin complex or three actins for the F-actin alone were generated. The symmetry expanded particles were subjected to masked 3D classification without image alignment (four classes, *T* = 40). For the actomyosin complex, classes with clear density of myosin were selected and subjected to a second round of masked 3D classification. Classes with clear myosin density comparable to that of actin, which indicates near 100% occupancy of myosin binding to F-actin, were selected. For F-actin alone, classes with clear actin density were retained after each round of 3D classification. Selected classes were used as input for a masked local refinement. Final postprocessing for each dataset produced density maps with resolution of 2.62 Å (F-actin alone), 3.10 Å (rigor wild-type myosin-15), 4.18 Å (rigor *jordan* mutant myosin-15), and 3.90 Å (ADP wild-type myosin-15) by the FSC 0.143 criterion (fig. S8). To separate the different conformations of the D-loop for F-actin alone and rigor wild-type myosin-15, spherical masks centering on the D-loop with gradually increased diameters were generated. The symmetry expanded particles from the last masked local refinement were subjected to masked 3D classification without image alignment (four classes, *T* = 100), using the converged map from the last refinement as the initial reference, low-pass–filtered to 3 Å. The mask with a diameter of 60 Å yields classes with clear and well-separated D-loop density.

To improve the resolution in the lever arm region of myosin-15 in the wild-type rigor and ADP states, particles from the final masked local refinement were reextracted after recentering on the second β strand of the transducer (S1245). Local refinement was performed with a mask of three actins and one myosin that extended to the first IQ motif and the associated light chain. The particles were then Bayesian polished and subjected to another round of masked local refinement. Final postprocessing yielded density maps of 3.17 Å (rigor wild-type myosin-15) and 4.15 Å (ADP wild-type myosin-15) by the FSC 0.143 criterion.

### Model building and structure refinement

For F-actin alone, a published actin model [Protein Data Bank (PDB): 6BNO] (*22*) was docked into the EM density by rigid body fitting in UCSF Chimera (*69*) and manually adjusted in Coot (*70*). In this starting model, the D-loop was modeled in the Out conformation. For the actomyosin structures, an initial homology model of the myosin-15 motor domain was generated with I-TASSER (*71*) using the crystal structure of myosin V (PDB: 1OE9) as the template. The myosin-15 model and the F-actin alone model were then docked into each cryo-EM density map to generate actomyosin starting models.

Models were then subjected to Rosetta density-guided model rebuilding (*72*). For each initial model, 200 models were generated, and the top 10 lowest energy models were manually inspected in Coot. Nonoverlapping stretches of amino acids that fit the cryo-EM density best were selected from these individual models then stitched together to build the full model. For F-actin alone and rigor wild-type actomyosin-15, the D-loop of a subset of the 10 lowest-energy models adopted the In conformation, which were subsequently used to model this conformer for these states. D-loops for all lowest-energy models in the *jordan* mutant reconstruction and wild-type ADP reconstruction adopted the In conformation. The stitched full models were refined using phenix.real_space.refine in the Phenix software package (*73*), iterated with manual adjustment in Coot. Symmetry expansion–based data processing generated additional clear density for the distal lever arm and the RLC. The lever arm model was manually extended. A homology model of chicken RLC (MYL12B) was created using the SWISS-MODEL (*74*) server with the AlphaFold (*75*) predicted human RLC (MYL12B) as the template (Identifier: AF-O14950-F1). The model was first rigid body–docked to the density map in UCSF ChimeraX and then flexibly fitted to the density with ISOLDE (*76*) using distance and torsion restrains for the secondary structure. The full model was refined using phenix.real_space.refine and manually adjusted in Coot. Model validation was conducted with MolProbity (*77*) as implemented in Phenix.

### Multisubunit F-actin model generation and superposition

The 31 subunit actin filament models for bare F-actin and wild-type rigor myosin-15–bound F-actin are generated by iteratively aligning multiple copies of the central three actin subunits (chains A, B, and D) from the models built above. Chains A and B are two adjacent subunits on the same protofilament and chain D binds to chains A and B from the other protofilament. For each alignment, chain A of the next model is aligned to chain B of the previous model, and then the overlapping chain A of the next model is removed. The resulting 31 subunit actin filaments are aligned on the first subunit at the barbed end.

### Molecular graphics and multiple sequence analysis

Figures and movies were generated with PyMOL (The PyMOL Molecular Graphics System, Version 2.3.4; Schrödinger LLC), UCSF Chimera (*69*), and ChimeraX (*78*). Multiple sequence alignment was performed with Clustal Omega (*79*) and formatted with Jalview (*80*).

### Pyrene actin polymerization

Skeletal rabbit actin was purified and labeled on C374 with *N*-(1-pyrene)-iodoacetamide. A correction factor was applied for determining pyrene actin concentration, *A*_corr_ = *A*_290_ – (0.127 × *A*_344_). Actin polymerization was measured in a cuvette-based fluorometer (PTI Quantamaster 400, HORIBA Scientific) with excitation at 365 nm and emission at 407 nm. Gel-filtered G-actin monomers (10% pyrene labeled) were desalted (PD SpinTrap G-25, Cytiva) into a modified G-buffer without ATP [2 mM tris-HCl, 0.1 mM CaCl_2_, 1 mM NaN_3_, and 1 mM DTT (pH 8.0)], stored on ice, and used within 3 hours. G-actin was converted to the Mg^2+^-bound form by addition of 50 μM MgCl_2_ and 0.2 mM EGTA for 2 min at room temperature, before starting the polymerization reaction by mixing G-actin (3× stock) with KMEI buffer (1.5× stock) in a 1:2 ratio, respectively. Wild-type myosin and ADP were included in the 1.5× KMEI buffer, as needed. Final reaction conditions were 2 μM G-actin, 1 μM myosin-15, 50 mM KCl, 1 mM MgCl_2_, 1 mM EGTA, and 10 mM imidazole (pH 7.0) at 25° ± 0.1°C. Data were corrected for the reaction dead time.

### Single-filament actin polymerization

Skeletal rabbit actin was purified, labeled on C374 using tetramethylrhodamine (TMR)-5-maleimide (Adipogen Life Sciences) and its concentration measured using *A*_corr_ = *A*_290_ – (0.208 × *A*_550_). Biotinylated skeletal muscle actin (#8109-01, Hypermol, Germany) was dialyzed against G-buffer and cleared by ultracentrifugation at 100,000*g* for 1 hour. G-actin stocks were prepared with TMR-actin (20%) and biotin-actin (10%) doping and desalted into a modified ATP-free G-buffer as described above. Cover glass (24 mm by 50 mm; #1.5, Marienfeld Superior, Germany) was sequentially sonicated in 2% Hellmanex III (Hellma, Germany), 1 M KOH, 100% ethanol, and, lastly, Milli-Q water, before drying under a nitrogen stream and processing under argon plasma (ZEPTO, Diener Electronic, Germany). Cleaned cover glass was coated with mPEG-silane (2 mg/ml; Laysan Bio, AL) and biotin–PEG–silane (2 μg/ml; Laysan Bio) diluted in 96% ethanol and 0.1% (v/v) HCl and baked at 70°C for 1 hour. Coverslips were rinsed in 96% ethanol and sonicated, followed by rinsing and sonication in Milli-Q water, and lastly dried under a filtered nitrogen stream. Flow chambers were assembled using double-sided sticky tape to create a channel on a glass slide. Flow cells were washed with T50 butter [10 mM tris-HCl, 50 mM KCl, and 1 mM DTT (pH 8.0)], incubated with neutravidin (0.1 mg/ml; Thermo Fisher Scientific) in T50 for 1 min, and then washed in bovine serum albumin (1 mg/ml; A0281, Sigma-Aldrich) in T50 for 30 s, followed by a final wash of T50.

Experiments were performed in the following buffer: 50 mM KCl, 1 mM MgCl_2_, 1 mM EGTA, 10 mM imidazole, 10 mM DTT, 15 mM glucose, 0.5% methylcellulose, catalase (20 μg/ml), and glucose oxidase (100 μg/ml) (pH 7.0) at 21° ± 1°C. Purified myosin-15 motor domain (1 μM) and ADP were included as needed. The reaction flow cell was imaged using an inverted microscope (Nikon Ti-E2) equipped with an oil immersion objective (CFI Apochromat TIRF 100×, 1.49 numerical aperture; Nikon) for objective-style TIRF microscopy (H-TIRF, Nikon). TMR fluorescence was excited using 561 nm and emission-filtered (ET630/75m, Chroma). Images were captured on an EM–charge-coupled device camera (iXon Ultra 888, Andor) controlled by NIS-Elements (AR version 5.2, Nikon). Data were corrected for the assay dead time. Images were background-subtracted and registered (descriptor-based series registration, 2d/3d + t) in FIJI (https://fiji.sc). Actin filament densities were quantified using the Analyze Particle command (size > 3 pixel^2^, circularity: 0.0 to 1.0) to count particles within a 50 μm by 50 μm region of interest that was randomly selected from the image. A minimum of three experiments, from two independent protein preparations, were analyzed for each condition. Filament elongation rates were calculated using kymographs generated in Elements software (Nikon).

### Statistical analysis

GraphPad Prism 9 (La Jolla, CA, USA) was used for statistical analysis of the data presented in Fig. 7 (details of statistical tests are presented in the figure legend). *P* values less than 0.05 were considered significant.

## Notes

View/request a protocol for this paper from *Bio-protocol*.
