# Supplementary material for: Structural basis for tunable control of actin dynamics by myosin-15 in mechanosensory stereocilia
Source: Sci Adv. 2022 Jul 20;8(29):eabl4733. doi: 10.1126/sciadv.abl4733 (PMC9299544; doi:10.1126/sciadv.abl4733)
Supplement: Supplementary file 1 — Figs. S1 to S11 Tables S1 and S2 [file sciadv.abl4733_sm.pdf]

Science Advances
AAAS

Supplementary Materials for

Structural basis for tunable control of actin dynamics by myosin-15 in mechanosensory stereocilia

Rui Gong *et al.*

Corresponding author: Jonathan E. Bird, j.bird@ufl.edu; Gregory M. Alushin, galushin@rockefeller.edu

Sci. Adv. **8**, eabl4733 (2022)
DOI: 10.1126/sciadv.abl4733

The PDF file includes:

Figs. S1 to S11
Tables S1 and S2
Legends for movies S1 to S6

Other Supplementary Material for this manuscript includes the following:

Movies S1 to S6

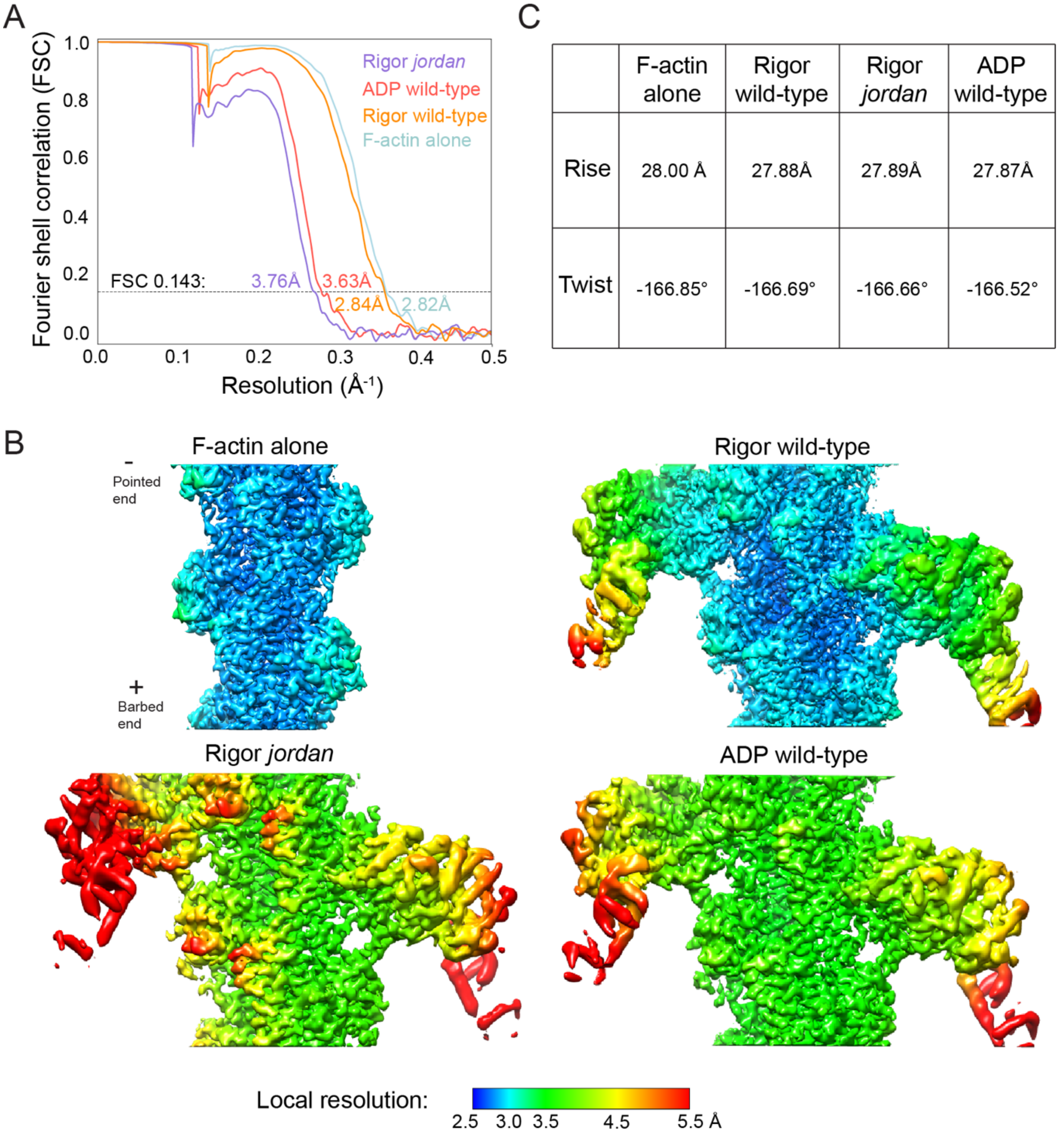

	F-actin alone	Rigor wild-type	Rigor *jordan*	ADP wild-type
Rise	28.00 Å	27.88Å	27.89Å	27.87Å
Twist	-166.85°	-166.69°	-166.66°	-166.52°

Fig. S1. Resolution assessment of cryo-EM reconstructions and helical parameters. (A) Gold-standard Fourier shell correlation (FSC) curves for all 3D reconstructions processed by implementing helical symmetry. Overall resolution is estimated by the FSC 0.143 criterion. **(B)** Local resolution estimation of the indicated reconstructions. **(C)** Helical parameters of the 3D reconstructions.

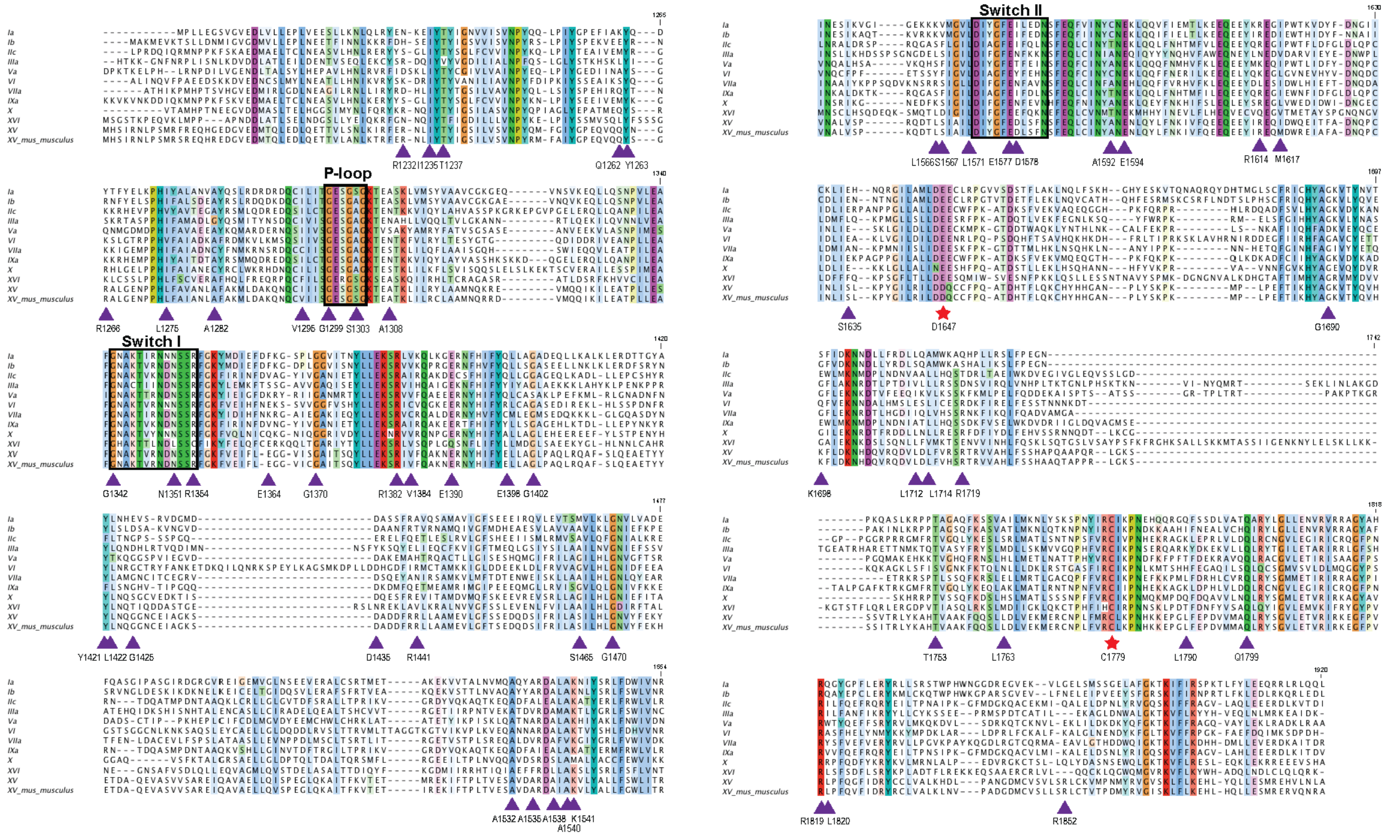

Fig. S2. Sequence alignment of myosin motor domains. Aligned sequences correspond to: *H. sapiens* myosin-1a (NP_005370.1), 1b (NP_001155291.1), 2c (NP_079005.3), 3a (NP_059129.3), 5a (NP_000250.3), 6 (NP_004990.3), 7a (NP_000251.3), 9a (NP_002464.1), 10 (NP_036466.2), 16 (NP_001185879.1), 15 (NP_057323.3), and *M. musculus* 15 (NP_874357). The alignment is colored by sequence conservation. The site of D1647 and C1779 are indicated by red stars, and sites of deafness mutations are indicated by purple triangles. Sequence alignment was performed using Clustal Omega and formatted with Jalview.

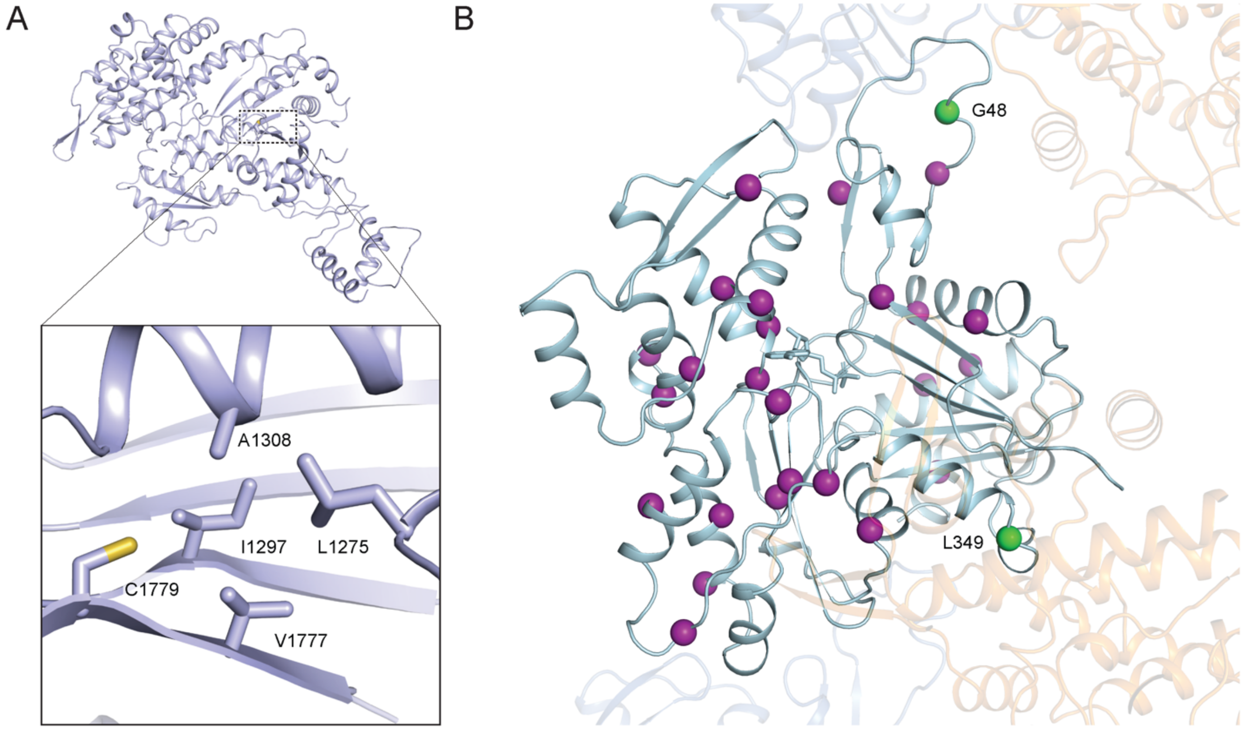

Fig. S3. Additional structural analysis of deafness causing mutations. (A) The hydrophobic environment surrounding C1779, the site of the *shaker 2* mutation. Hydrophobic residues are shown as sticks. **(B)** Deafness causing mutations on γ-actin shown as magenta spheres. Two residues located at the actomyosin-15 interface are highlighted in green. Adjacent actin subunits and myosin-15 are shown in transparent blue and orange, respectively.

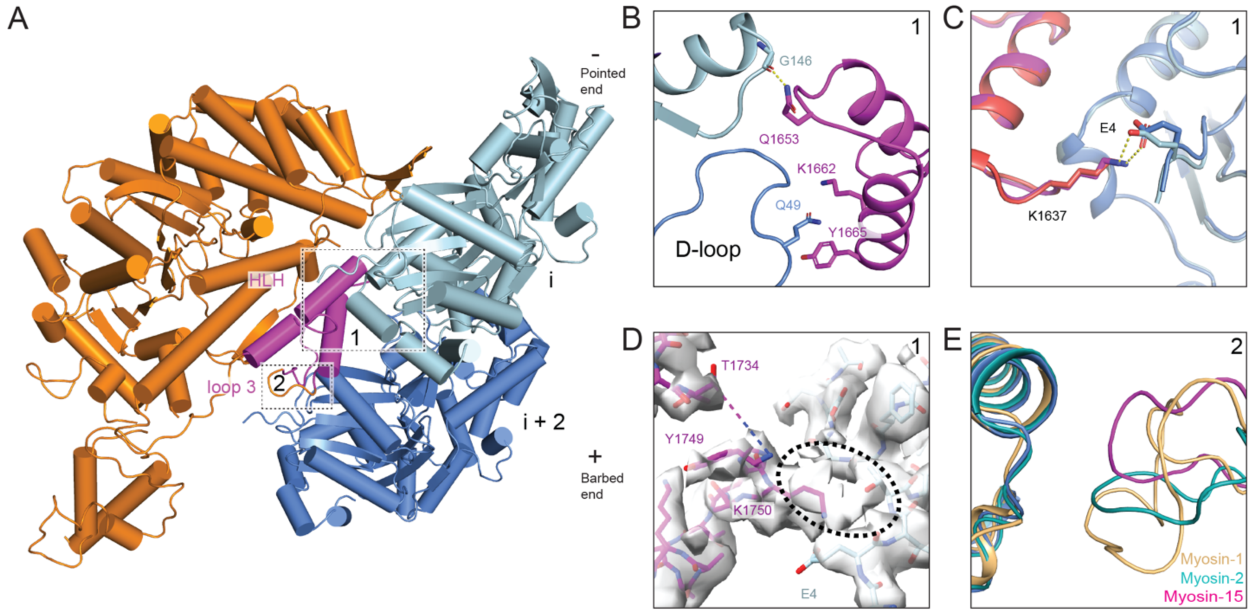

Fig. S4. Additional interactions at the actomyosin-15 interface. (A) Atomic model of the interface between two actin subunits and rigor wild-type myosin-15 shown from an alternative perspective. Actin subunits i, i+2, and myosin-15 are colored in light blue, cornflower blue, and dark orange, respectively. The indicated actin interaction motifs are highlighted in magenta, and numbered boxes correspond to detail views in subsequent panels. **(B)** Electrostatic interactions between the HLH of rigor wild-type myosin-15 and F-actin. **(C)** Interaction between the activation loop of myosin-15 and the N-terminus of actin subunit i. The HLH of the rigor and ADP wild-type myosin-15 are colored in magenta and deep salmon. The corresponding actin subunits are colored in light blue and cornflower blue. **(D)** The density map around loop 2 of rigor wild-type myosin-15. The model of myosin-15 and actin are colored in magenta and light blue. The disordered region of loop 2 between residues T1734 and Y1749 is represented by a dashed line. Extra density near the N terminus of actin is circled. **(E)** Structural comparison of the myosin loop 3-actin interface between rigor state myosin-15, myosin-1b, and myosin-2. The structures are superimposed on the loop 3-interacting actin subunit. Loop 3 of myosin-15 is colored in magenta and the interacting actin is colored in cornflower blue, while loop 3 of myosin-1b and myosin-2c are colored in light orange and teal, respectively, as are their interacting actin subunits.

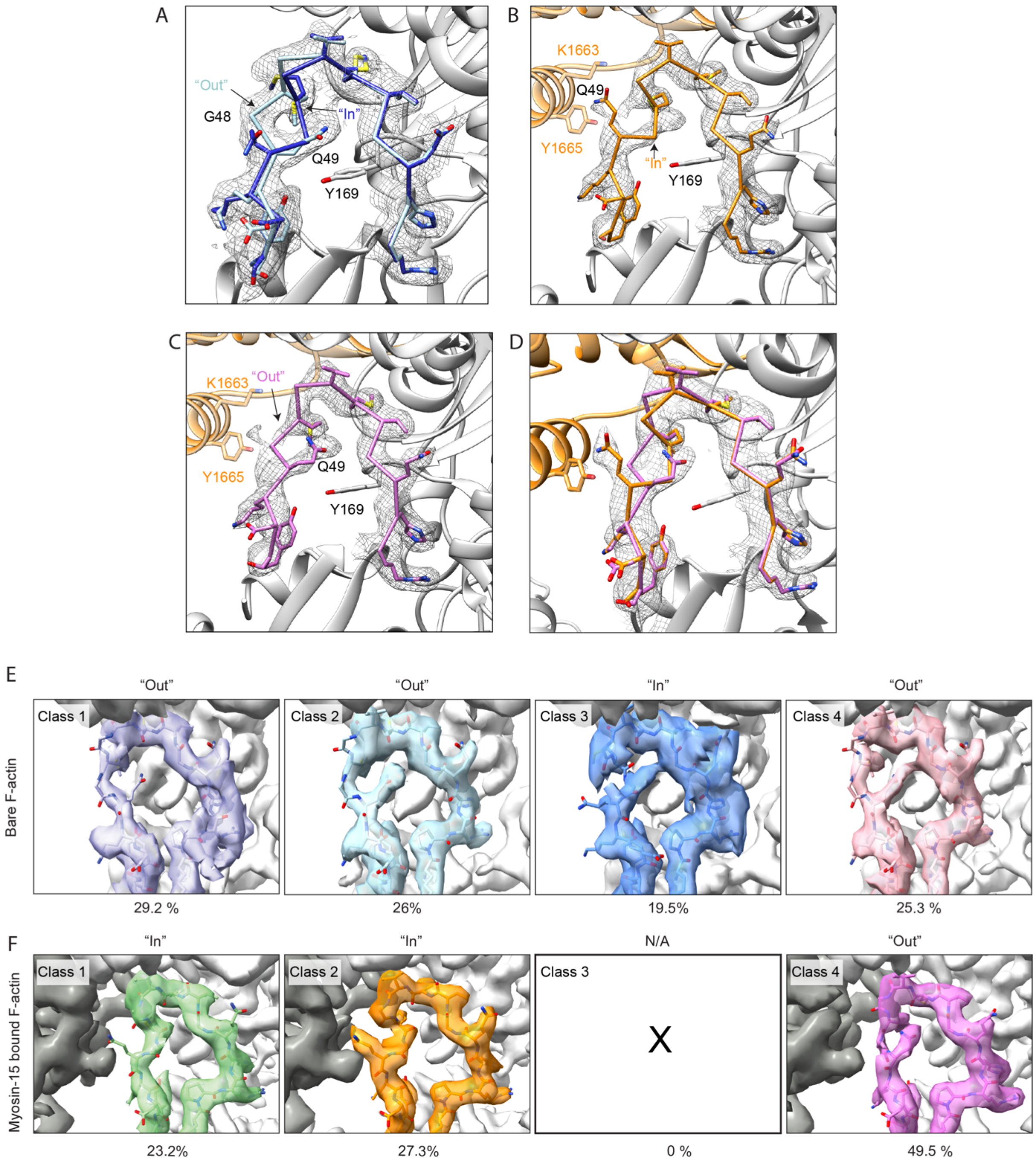

Fig. S5. Myosin-15 binding modulates D-loop flexibility in F-actin. (A) Segmented cryo-EM density of the bare F-actin D-loop rendered at 0.025 RMS, superimposed with stick models of the "Out" and "In" D-loop conformations colored in light blue and medium blue, respectively. (**B** and **C**) Segmented cryo-EM density of the rigor wild-type myosin-15 bound actin D-loop, superimposed with stick models of the "In" (dark orange, B) and "Out" (pink, C) D-loop conformations, respectively. (**D**) Segmented cryo-EM density of the rigor wild-type myosin-15 bound actin D-loop after symmetry expansion, superimposed with stick models of the "Out" and "In" D-loop conformations, respectively. (**E**) Segmented density maps of all four 3D classes from focused 3D classification of the bare F-actin dataset. D-loop densities are highlighted and

superimposed with stick models of the “Out” or “In” D-loop conformations. (**F**) Segmented density maps of all three 3D classes from the focused 3D classification of the rigor wild-type myosin-15 bound F-actin dataset. D-loop densities are highlighted and superimposed with stick models of the “Out” or “In” D-loop conformations.

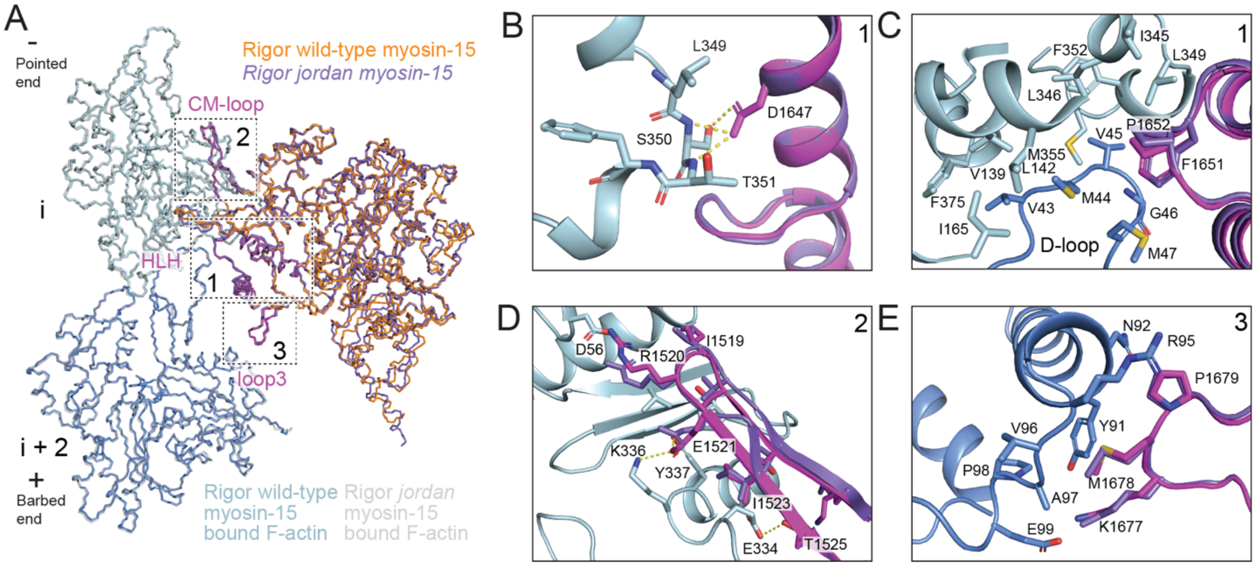

Fig. S6. Structural comparison of rigor wild-type and *jordan* mutant actomyosin-15 at the actin-myosin interface. (A) Backbone representations of the rigor wild-type and *jordan* mutant actomyosin-15 models, superimposed on actin subunit i. Myosin-15's actin interacting motifs are highlighted in magenta on the rigor wild-type model. Numbered boxes correspond to detail views in subsequent panels, which match those displayed in Figure 3. **(B)** Although the D1647G lesion disrupts interactions in the *jordan* mutant, the overall HLH conformation in this region is maintained. **(C-E)** All other actin interface interactions mediated by the HLH (**C**), CM loop (**D**), and loop 3 (**E**), are maintained in the *jordan* mutant.

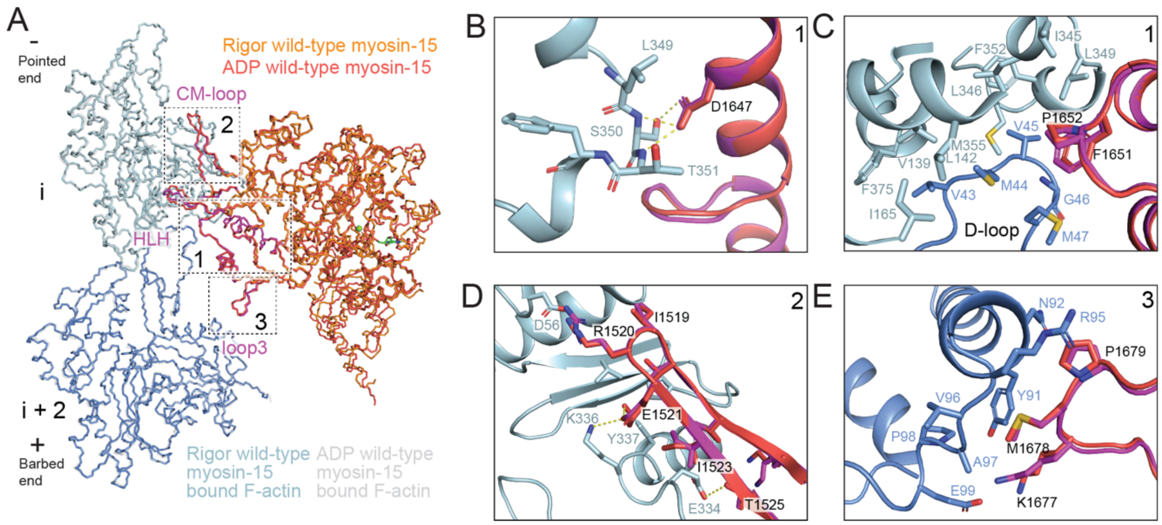

Fig. S7. Structural comparison of rigor and ADP wild-type actomyosin-15 at the actin-myosin interface. (A) Backbone representations of the rigor and ADP wild-type actomyosin-15 models, superimposed on actin subunit i. Myosin-15's actin interacting motifs are highlighted in magenta on the rigor wild-type model. Numbered boxes correspond to detail views in subsequent panels, which match those displayed in Figure 3. **(B-E)** All actin interface interactions mediated by HLH residues D1647 (**B**) and F1651/P1652 (**C**), as well as the CM loop (**D**) and loop 3 (**E**), are present in both the ADP and rigor states.

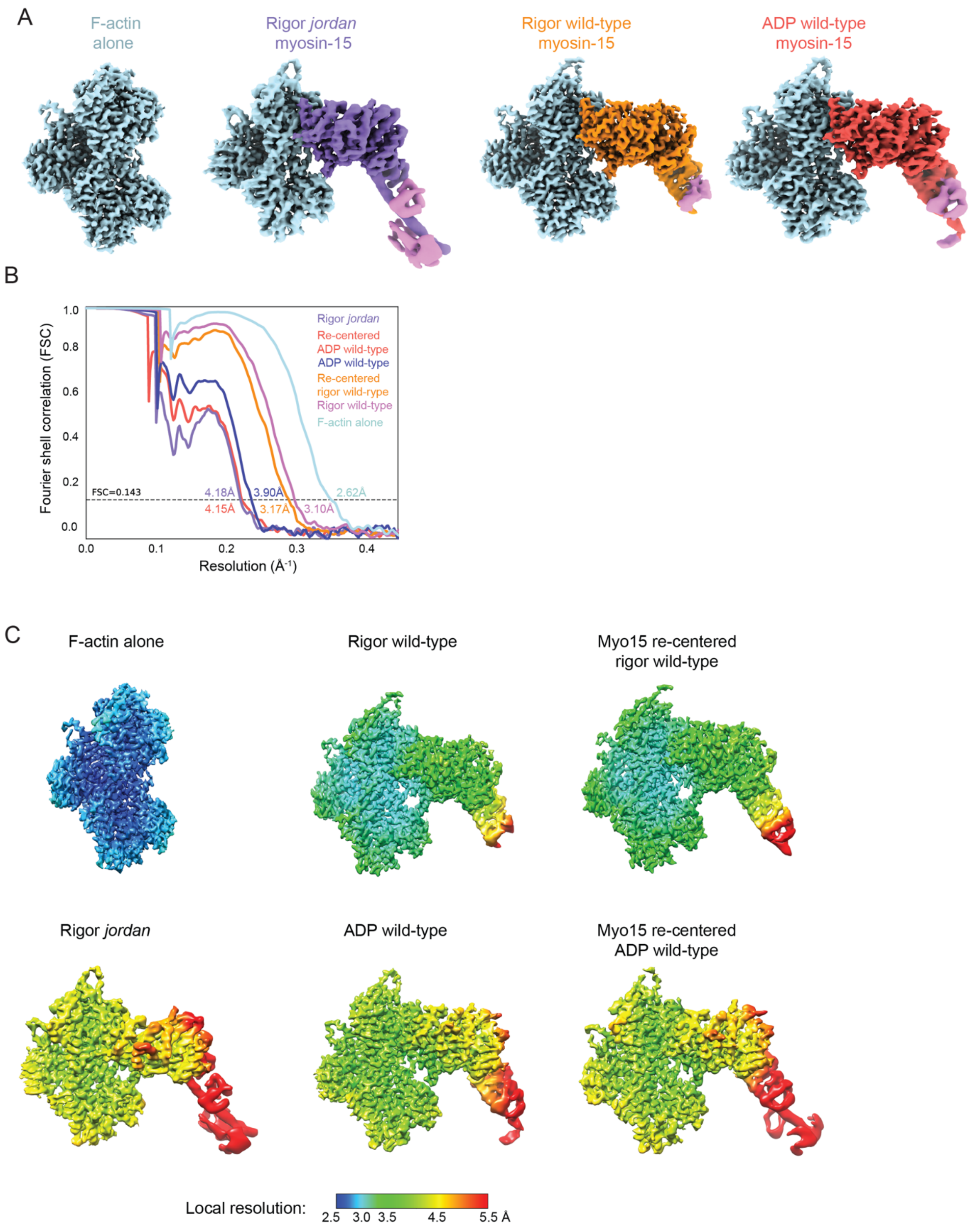

Fig. S8. Resolution assessment of cryo-EM reconstructions performed with symmetry expansion. (A) Cryo-EM maps of bare F-actin and actomyosin-15 complexes derived from symmetry expansion-based reprocessing. **(B)** Gold-standard Fourier shell correlation (FSC) curves for all 3D reconstructions reprocessed by implementing symmetry expansion. Overall resolution is estimated by the FSC 0.143 criterion. **(C)** Local resolution estimation of the indicated reconstructions.

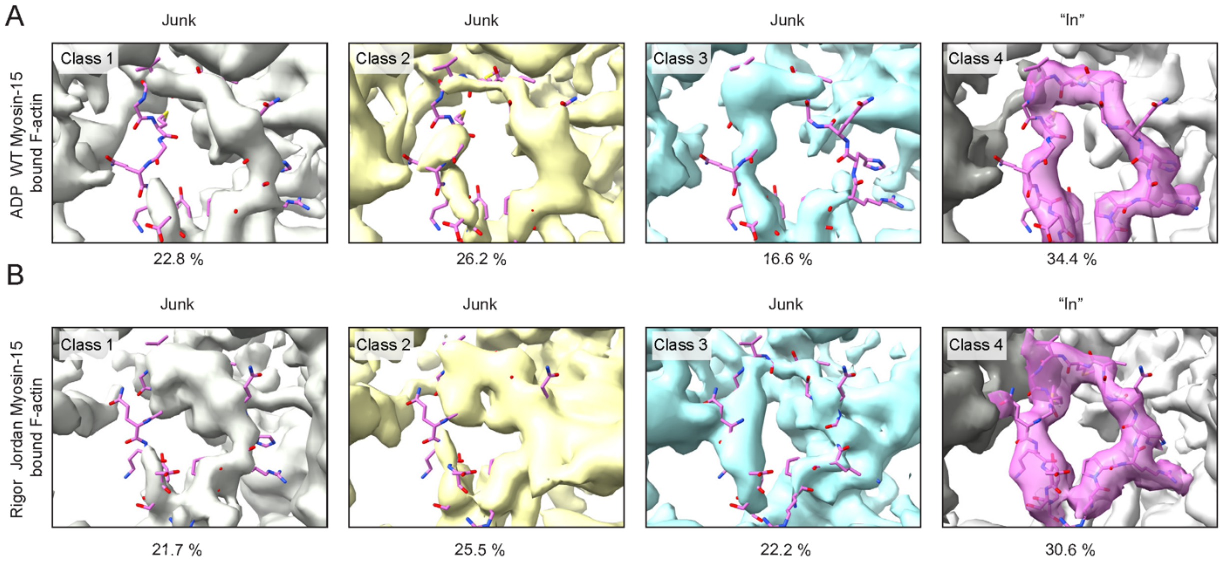

Fig. S9. Rigor *jordan* mutant and ADP bound wildtype myosin-15 lock the D-loop in the "In" conformation. (A and B) Segmented density maps of all four 3D classes from the focused 3D classification of the ADP state wild-type (A) and rigor state *jordan* (B) myosin-15 bound F-actin datasets, respectively. D-loop densities are shown and superimposed with stick models of the "In" D-loop conformations.

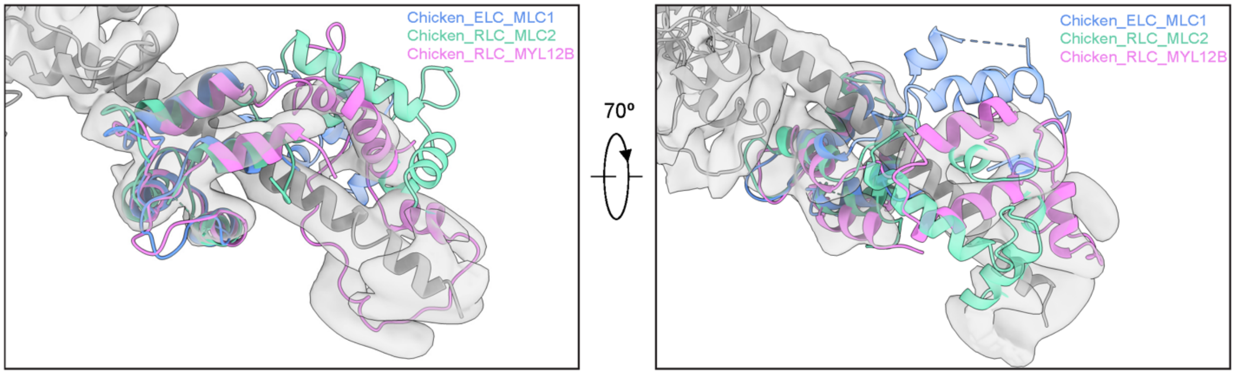

Fig. S10. Myosin15's regulatory light chain exhibits a unique structural arrangement. Chicken muscle essential light chain and regulatory light chains (PDB: 2MYS), colored in cornflower blue and aquamarine, respectively, are superposed on the regulatory light chain of myosin-15 colored in magenta. The convertor and lever arm of myosin-15 are colored in grey and the segmented density map of myosin-15 is displayed in transparent representation.

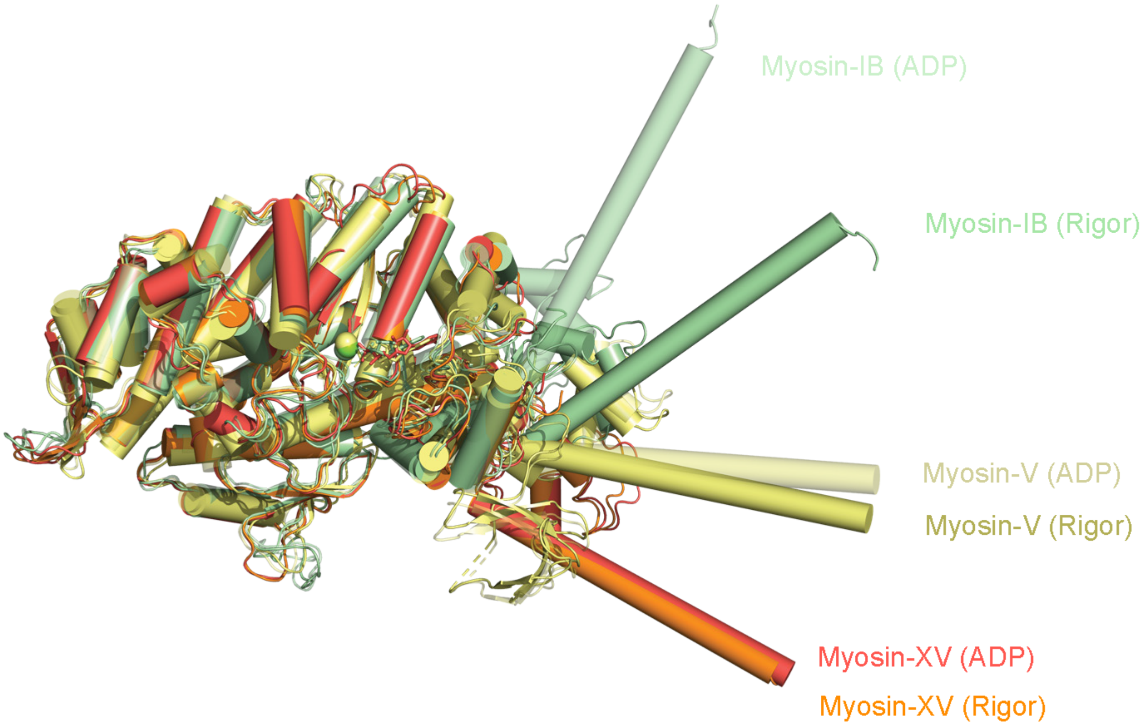

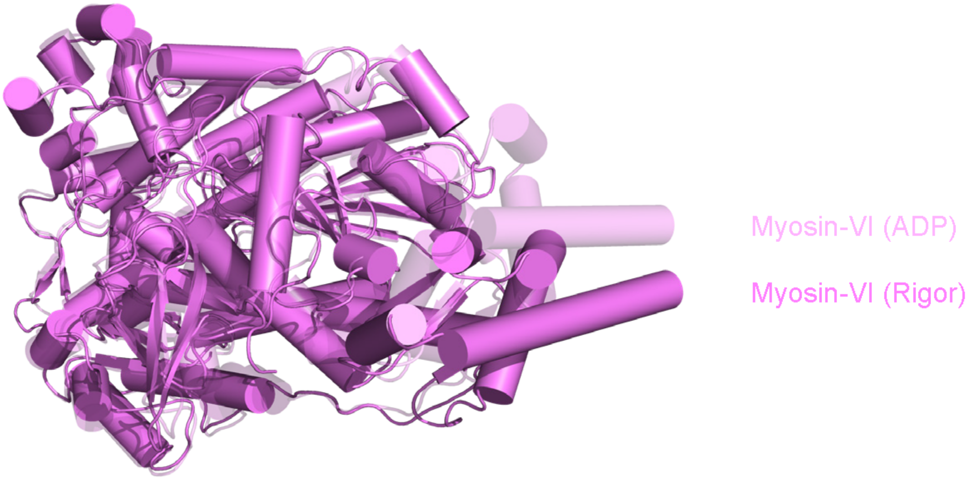

Fig. S11. Structural comparison of various F-actin bound myosins in the ADP and rigor state. Atomic models of the rigor and strong ADP state myosin-15 are colored in light orange and red. The rigor state myosin-1b (PDB: 6C1H), myosin V (PDB: 7PLT) and myosin VI (PDB:6BNV) are colored in pale green, pale yellow and violet, respectively. The strong ADP state myosin-1b (PDB: 6C1D), myosin V (PDB: 7PM5) and myosin VI (PDB:6BNW) are shown in transparent representation. Light chains are omitted for clarity.

	F-actin alone (EMD-24321, PDB 7R8V)	Myosin-15 (WT-rigor) bound F-actin (EMD-24322, PDB: 7R91)	Myosin-15 (Jordan-rigor) bound F-actin (EMD-24400, PDB: 7RB9)	Myosin-15 (WT-ADP) bound F-actin (EMD-24399, PDB: 7RB8)
**Data collection and processing**				
Microscope	Titan Krios	Titan Krios	Titan Krios	Titan Krios
Voltage (kV)	300	300	300	300
Detector	K2 Summit	K2 Summit	K2 Summit	K2 Summit
Magnification	29,000	29,000	29,000	29,000
Electron exposure (e^- / Å^2)	60	60	60	60
Exposure rate (e^- / pixel / s)	6	6	6	6
Calibrated pixel size (Å)	1.03	1.03	1.03	1.03
Defocus range (μm)	–1.5 to –3.5	–1.5 to –3.5	–1.5 to –3.5	–1.5 to –3.5
Helical symmetry	C1	C1	C1	C1
	28.00 Å rise	27.88 Å rise	27.89 Å rise	27.87 Å rise
	-166.85° twist	-166.69° twist	-166.66° twist	-166.52° twist
Initial filament segments (no.)	229833	150424	113662	139547
Selected filament segments after 2D classification (no.)	228334	142635	91340	125232
Selected filament segments after 3D classification (no.)	228334	142635	91340	125232
Final filament segments (no.)	228334	142635	91340	125232
Map resolution (Å)	2.82	2.83	3.76	3.63
FSC threshold	0.143	0.143	0.143	0.143
**Refinement**				
Initial model (PDB ID)	6BNO	6BNO	6BNO	6BNO
Model resolution (Å)	3.0	3.0	3.9	3.8
FSC threshold	0.5	0.5	0.5	0.5
Map sharpening B factor (Å^2)	–44.79	-50.05	-86.65	-84.62
Model composition	5 actin protomers	3 actin protomers, 1 myosin 15	3 actin protomers, 1 myosin 15	3 actin protomers, 1 myosin 15
Non-hydrogen atoms	14845	14320	14193	14226
Protein residues	1855	1786	1786	1786
Ligands	5 Mg.ADP	3 Mg.ADP	3 Mg.ADP	4 Mg.ADP
B factors (Å^2)				
Protein	37.16	54.6	120.24	79.79
Ligand	27.66	29.25	77.38	70.69
R.M.S. deviations				
Bond lengths (Å)	0.004	0.004	0.004	0.004
Bond angles (°)	0.97	0.978	0.979	7.018
**Validation**				
MolProbity score	1.44	1.37	1.7	1.84
Clash score	5.56	5.01	9.24	8.73
Poor rotamers (%)	0	0.19	0	0.07
Ramachandran plot				
Favored (%)	97.27	97.45	96.67	94.62
Allowed (%)	2.73	2.55	3.33	5.38
Disallowed (%)	0.00	0.00	0.00	0.00

Table S1. Cryo-EM data collection, refinement and validation statistics for reconstructions performed with helical symmetry

	F-actin alone (EMD-26463)	WT-rigor Myo15 bound F-actin (EMD-26462)	ADP Myo15 bound F-actin (EMD-26461)	Jordan Myo15 bound F-actin (EMD-26464)	Re-centered F-actin bound WT-rigor Myo15 (EMD-26459, PDB:7UDT)	Re-centered F-actin bound WT-ADP Myo15 (EMD-26460, PDB: 7UDU)
**Data collection and processing**						
Microscope	Titan Krios					
Voltage (kV)	300					
Detector	K2 Summit					
Magnification	29,000					
Electron exposure (e^- / Å^2)	60					
Exposure rate (e^- / pixel / s)	6					
Calibrated pixel size (Å)	1.03					
Defocus range (µm)	–1.5 to –3.5					
Helical symmetry expansion parameters	C1					
	28.00 Å rise	27.88 Å rise	27.87 Å rise	27.89 Å rise	27.88 Å rise	27.87 Å rise
	-166.85° twist	-166.69° twist	-166.52° twist	-166.66° twist	-166.69° twist	-166.52° twist
Initial symmetry expanded particle images (no.)	577317	427905	375696	274020	427905	375696
Final symmetry expanded particle images (no.)	425542	223793	189104	232001	223793	189104
Map resolution (Å)	2.62	3.10	3.9	4.18	3.17	4.15
FSC threshold	0.143					
**Refinement**						
Initial model (PDB ID)					7R91	7RB8
Model resolution (Å)					3.4	4.2
FSC threshold					0.5	0.5
Map sharpening B factor (Å^2)					-58.21	-110.4
Model composition					3 actin protomers, 1 myosin 15	3 actin protomers, 1 myosin 15
Non-hydrogen atoms					15806	15711
Protein residues					1966	1966
Ligands					3 Mg.ADP	4 Mg.ADP
B factors (Å^2)						
Protein					77.94	163.33
Ligand					70.98	190.74
R.M.S. deviations						
Bond lengths (Å)					0.003	0.003
Bond angles (°)					0.587	0.672
**Validation**						
MolProbity score					1.62	1.94
Clash score					7.32	10.35
Poor rotamers (%)					0.24	0.06
Ramachandran plot						
Favored (%)					96.65	93.98
Allowed (%)					3.35	6.02
Disallowed (%)					0.00	0.00

Table S2. Cryo-EM data collection, refinement and validation statistics for reconstructions performed with symmetry expansion

Movie S1: Morph of segmented actin density between F-actin alone and rigor wild-type myosin-15 bound F-actin. Maps were lowpass filtered to 5 Å.

Movie S2: Detail view of D-loop in morph between F-actin alone and rigor wild-type myosin-15 bound F-actin. Maps are identical to those presented in Movie S1.

Movie S3: Morph of segmented actin density between rigor wild-type and *jordan* myosin-15 bound F-actin. Maps were lowpass filtered to 5 Å.

Movie S4: Detail view of D-loop in morph between rigor wild-type and *jordan* myosin-15 bound F-actin. Maps are identical to those presented in Movie S3.

Movie S5: Morph of segmented actin density between rigor and ADP wild-type myosin-15 bound F-actin. Maps were lowpass filtered to 5 Å.

Movie S6: Detail view of D-loop in morph between rigor and ADP wild-type myosin-15 bound F-actin. Maps are identical to those presented in Movie S5.
